# Skeletal dysmorphology and mineralization defects in Fgf20 KO mice

**DOI:** 10.3389/fendo.2024.1286365

**Published:** 2024-07-26

**Authors:** Sylvie Dlugosova, Frantisek Spoutil, Carlos Eduardo Madureira Trufen, Betul Melike Ogan, Michaela Prochazkova, Olha Fedosieieva, Petr Nickl, Goretti Aranaz Novaliches, Radislav Sedlacek, Jan Prochazka

**Affiliations:** ^1^ Czech Centre for Phenogenomics, Institute of Molecular Genetics of the Czech Academy of Sciences, Vestec, Czechia; ^2^ Laboratory of Transgenic Models of Diseases, Institute of Molecular Genetics of the Czech Academy of Sciences, Vestec, Czechia

**Keywords:** Fgf, mineralization, bone, chondrocytes, bone homeostasis, polydactylia

## Abstract

**Introduction:**

Fibroblast growth factor 20 (Fgf20), a member of the Fgf9 subfamily, was identified as an important regulator of bone differentiation and homeostasis processes. However, the role of Fgf20 in bone physiology has not been approached yet. Here we present a comprehensive bone phenotype analysis of mice with functional ablation of Fgf20.

**Methods:**

The study conducts an extensive analysis of Fgf20 knockout mice compared to controls, incorporating microCT scanning, volumetric analysis, Fgf9 subfamily expression and stimulation experiment and histological evaluation.

**Results:**

The bone phenotype could be detected especially in the area of​ the lumbar and caudal part of the spine and in fingers. Regarding the spine, Fgf20^-/-^ mice exhibited adhesions of the transverse process of the sixth lumbar vertebra to the pelvis as well as malformations in the distal part of their tails. Preaxial polydactyly and polysyndactyly in varying degrees of severity were also detected. High resolution microCT analysis of distal femurs and the fourth lumbar vertebra showed significant differences in structure and mineralization in both cortical and trabecular bone. These findings were histologically validated and may be associated with the expression of Fgf20 in chondrocytes and their progenitors. Moreover, histological sections demonstrated increased bone tissue formation, disruption of Fgf20^-/-^ femur cartilage, and cellular-level alterations, particularly in osteoclasts. We also observed molar dysmorphology, including root taurodontism, and described variations in mineralization and dentin thickness.

**Discussion:**

Our analysis provides evidence that Fgf20, together with other members of the Fgf9 subfamily, plays a crucial regulatory role in skeletal development and bone homeostasis.

## Introduction

Fibroblast growth factors (Fgfs) are a family of signalling factors consisting of 7 subfamilies of Fgf ligands encoded by 22 different genes (1) whose signalling pathway is mediated by the receptor tyrosine kinases Fgfr1–Fgfr4 (2). The specification and fine tuning of Fgf signalling in a strictly regulated temporo-spatial manner is achieved by alternative Fgf receptor mRNA splicing and distinct ligand binding properties of Fgf ligands (3).

Fgfs play fundamental pleiotropic roles such as stimulating cell proliferation, regulation of the differentiation process and organ morphogenesis (4, 5). Thus, Fgf signalling is involved in a plethora of developmental and physiological regulatory networks, i.e. in the lungs (6), pancreas (7), heart (8), kidney (9), craniofacial development (10) and others. One of the most important roles of Fgf signalling is in bone development and mineralization process. Fgf signalling is involved in mesenchymal condensation during embryonic development and also provides a critical signal for progenitor cell differentiation, growth plate regulation and mineral homeostasis in mature bone. Fgf signalling has important clinical implications in bone pathological conditions. Aberrant Fgf signalling leads to premature cranial suture fusions and premature termination of proliferation of growth plate chondrocytes in achondrodysplasia or Crouzon syndrome. The thorough review of Fgf signalling in bone can be found in Ornitz & Marie 2019 (11).

Fgf20 belongs to the Fgf9 subfamily along with Fgf9 and Fgf16 and, like the rest of the subfamily, recognizes the IIIc splicing variants of Fgfr3, Fgfr1 and Fgfr2. Fgfr4 and Fgfr3IIIb interact with a milder affinity (3, 12). Fgf20 is considered a key paracrine neurotrophic factor, also important for the survival of dopaminergic neurons (13). Therefore, a Fgf20 polymorphism is recognized as a Parkinson’s disease risk factor (14). During embryonic development, Fgf20 and Fgf9 show an overlapping expression pattern in cranial sutures as well as in the limb bud. The expression pattern suggests that there might be functional redundancy between Fgf9 and Fgf20 (15). A specific role of Fgf20 has been revealed during inner ear development. In cochlea, Fgf20 plays a major role in cellular differentiation in the lateral cochlear compartment within the Corti organ (16). Fgf20 has also been shown to be the main downstream effector of the Ectodysplasin (Eda) signalling pathway in ectodermal organs. In hair follicles, Fgf20 signalling is required for directing cell movement and forming dermal cell condensation (17, 18). In another ectodermal derivate, the tooth, Fgf20 is further expressed during the development up to the bell stage. Fgf20 expression is overlapping with that of other Fgf ligands situated at the primary enamel knot area and is part of the complex Fgf regulatory interaction between the epithelium and the underlying mesenchyme (19). Interestingly, a similar contribution to epithelial-mesenchymal interaction is described in the budding morphogenesis of mammary glands (20). The evidence of Fgf20’s role in bone mineralization was suggested from a large scale study of collaborative cross experiments (21) and also by similarity with the rest of Fgf9 subfamily; however, the clear involvement of Fgf20 in bone mineralization homeostasis has not been investigated yet (22).

Thus, we focused on detailed analysis of mineral tissues in Fgf20 KO mice, generated under the umbrella of the IMPC effort for whole genome annotation. We used high-resolution microCT scanning and volumetric analysis to compare the bone ultrastructure between Fgf20 KO mice and WT controls. Our findings clearly demonstrate that Fgf20 plays a nonredundant role not only in skeletal bone mineralization and homeostasis but also in dental structures of molars. We found deviations in cortical bone architecture and, more importantly, also in trabecular bone density. Those finding were furthermore studied in detail on histological sections, were confirmed and, additionally, revealed pathological changes in articular cartilage and alterations at the histological level. Our results prove that the Fgf20 gene variants can be considered a skeletal mineralization risk factor, as previously suggested. This knowledge could have implications for future research in bone-related pathologies, providing a foundation for developing targeted therapeutic interventions or preventive measures related to skeletal health and mineralization.

## Results

### Fgf20 ablation causes skeletal dysmorphology phenotype

**Figure 1:**
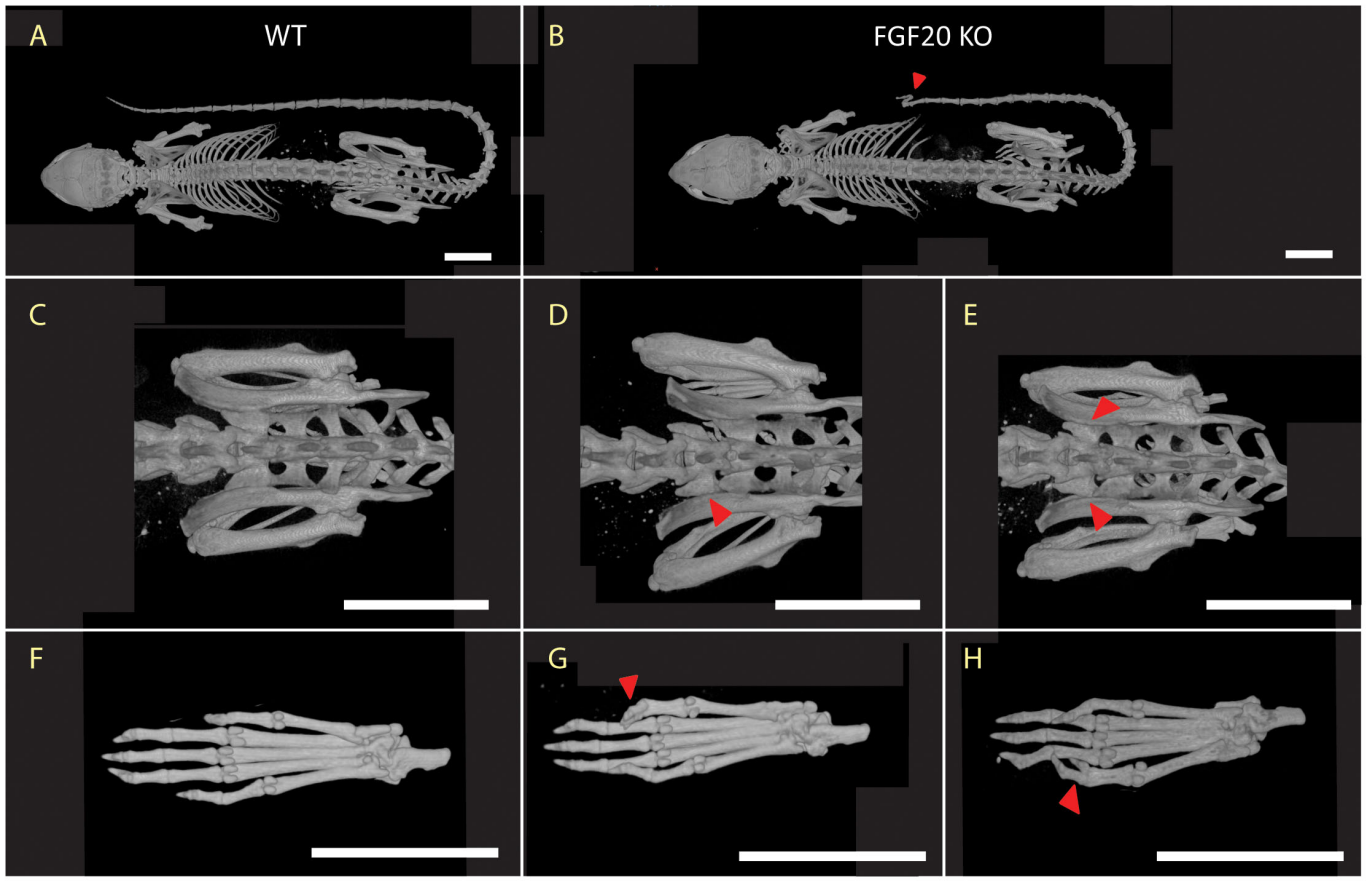
Skeletal morphology. Presentation of most common dysmorphologies in Fgf20 KO mutans **(B, D, E, G, H)**, highlighted with red arrowhead, and their comparison with standard morphology of WT **(A, C, F)**. The whole-body skeleton from the dorsal view **(A, B)** shows deformity in tail spine in Fgf20 KO mutant with a complete penetrance (12/12). Detail of pelvic region **(C–E)** shows deviation in transversal processes of L6 in Fgf20 KO mutant and its cooption to the sacral region – one-sided **(D)** males: 3/7) and two-sided **(E)** females: 1/5, males: 3/7). Skeleton of hind paws **(F–H)** showing polysyndactyly **(G)** females: 3/5) and Wassel type II. polydactyly **(H)** males: 1/7) found in Fgf20 KO mutants. Bar = 10 mm.

For detailed analysis of skeletal physiology, we used whole body microCT scans according to IMPC Impress procedures (23). The skeletal morphology defects were localized in the lumbar and caudal parts of the spine (**Figures 1A–E**). Fully penetrant malformations of the distal tail tip were observed in all Fgf KO mice (**Figure 1B**). We observed adhesion of the sixth lumbar vertebra (L6) to the pelvis by either a single transverse process (**Figure 1D**) or both (**Figure 1E**), resulting in gaining sacral vertebra morphology, and thus a change in segment identity. In three males (3/7), L6 exhibited partial fusion to the pelvis, while in one female (1/5) and three other males (3/7), L6 underwent complete fusion.

Besides the axial skeleton, there was a partly penetrant phenotype in the digit (3/5) in females with milder to fully developed polysyndactyly on the right hind paw (**Figures 1F, G**). We identified Wassel type II polydactyly with a duplicated distal phalanx on the left hind paw in males (**Figure 1H**), although with very low penetrance (1/7). These observations suggest a vital role of Fgf20 in digit development and, most likely, digit identity regulation.

### Fgf20 expression and a signalling activity

**Figure 2:**
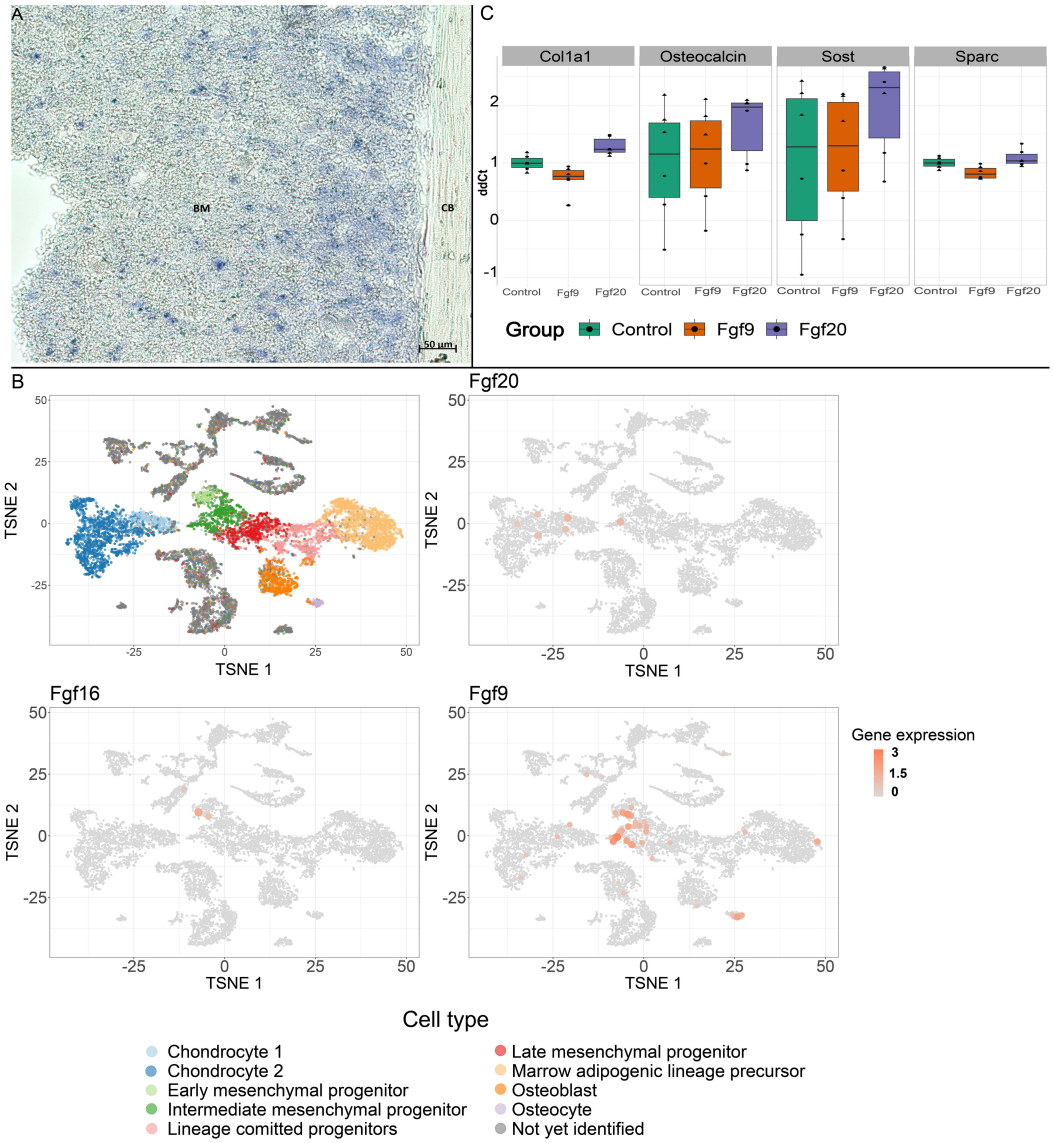
Expression of the Fgf20. **(A)** RNA *in situ* hybridization in long bone. Sections of the femoral diaphyses were processed for ISH with DIG-labeled Fgf20 probe. Note the dispersed signal. BM, bone marrow; CB, cortical bone. Scale bar shown (50 µm). **(B)** Expression of the Fgf9 subfamily members, Fgf20, Fgf16 and Fgf9, among distinct cell populations. Fgf20 expression is localized in clusters of intermediate mesenchymal progenitors and differentiated chondrocytes. Fgf16 expression is then localized in cells from early mesenchymal progenitor clusters while Fgf9 is enriched in the early, intermediate and late mesenchymal progenitors, chondrocytes, osteocytes and marrow adipogenic lineage precursors. **(C)** The effects of FGF9 and FGF20 on the mRNA expression levels of Col1a1, Osteocalcin, Sost and Sparc in MC3T3-E1 cells (see **Table 1** for p-values).

RNA *in situ* hybridization revealed a diffuse pattern of Fgf20 expression in sections of femur, permeating throughout the bone tissue (**Figure 2A**). While the exact localization of expression has not been ascertained, it holds functional potential in establishing a cell differentiating environment. Detailed image of highly positive cells and negative control staining with sense probe is shown in **Supplementary Figure S1** in the **Supplementary Material**.

For detailed expression analysis and specific mapping, we used publicly available single-cell sequencing datasets, particularly focusing on the stromal mesenchymal cells dataset (24). We analyzed the expression of all three Fgf members of the Fgf9 subfamily. Fgf20, Fgf16, and Fgf9 expression were interestingly distributed across distinct cell clusters (**Figure 2B**). Overall, the level of expression is low; however, the bone phenotype in Fgf9 heterozygote (25) and Fgf16 KO (26) was previously described. Fgf16 expression is localized in cells from early mesenchymal progenitor clusters, while Fgf9 is, moreover, enriched in intermediate and late mesenchymal progenitors, osteocytes, chondrocytes, and marrow adipogenic lineage precursors (**Figure 2B**). Contrary to Fgf9 and Fgf16, besides intermediate mesenchymal progenitors, Fgf20 is also localized in clusters of differentiated chondrocytes (**Figure 2B**). The distinct expression patterns of individual Fgf9 subfamily ligands suggest importance in expression regulation and lineage specificity.

To further investigate the signaling activity of the Fgf9 subfamily, we stimulated both Fgf20 and Fgf9 in the MC3T3-E1 pre-osteoblastic cell line. Subsequently, qPCR revealed heightened regulation in distinct osteoblast differentiation markers. Fgf9 exhibited a significant regulation of *Col1a1* and *Sparc*. Conversely, Fgf20 demonstrated a significantly regulated expression of *Col1a1*, *Osteocalcin*, and *Sost* with increased potency (**Table 1**, **Figure 2C**).

**Table 1:** The effect of FGF9 and FGF20 on mRNA expression levels of Sparc, Sost, Osteocalcin, Col1a1 and Alpl in MC3T3-E1 cells.

Gene	Comparison	Difference	p-value
Sparc	Fgf9 - Control	-0.17	0.01
Sparc	Fgf20 - Control	0.09	0.30
Sost	Fgf9 - Control	0.17	0.85
Sost	Fgf20 - Control	0.97	0.01
Osteocalcin	Fgf9 - Control	0.11	0.89
Osteocalcin	Fgf20 - Control	0.66	0.03
Col1a1	Fgf9 - Control	-0.28	0.01
Col1a1	Fgf20 - Control	0.29	0.01
Alpl	Fgf9 - Control	0.17	0.86
Alpl	Fgf20 - Control	0.21	0.79

The qPCR replicates were averaged, and delta Cq values were obtained by subtracting the reference gene Rpl19 from the gene of interest. The data were analyzed by averaging qPCR replicates, determining delta Cq values by subtracting the reference gene Rpl19 from the gene of interest. Subsequently, the mean of the control samples was subtracted from each sample to calculate delta-delta Cq. These values were then recalculated to relative values, with the mean of control samples set to 1. Significant results (p < 0.05) in red.

### Fgf20 ablation causes dysregulation of mineral homeostasis in bones

**Figure 3:**
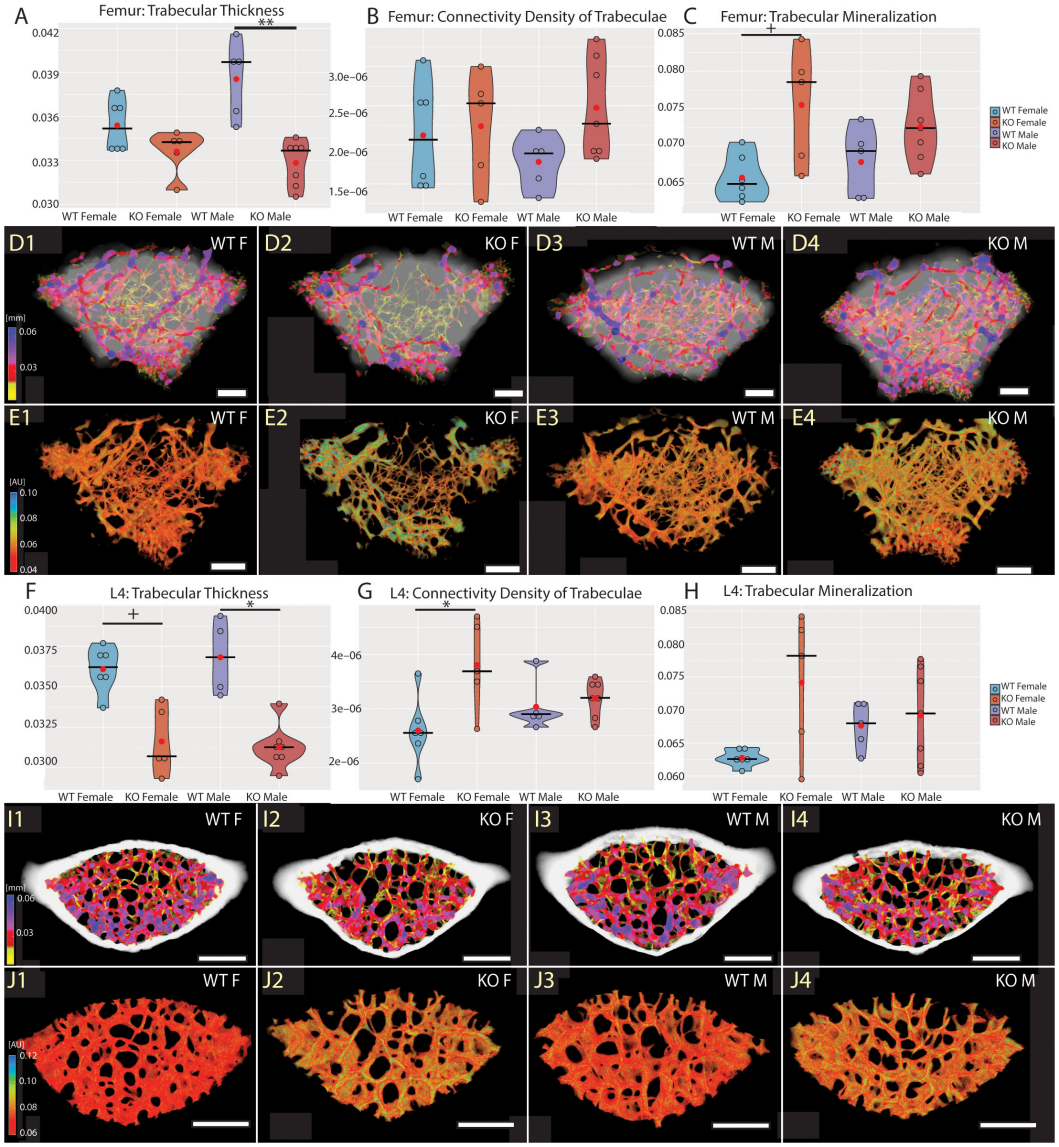
Trabecular bone of distal femur **(A–E)** and L4 vertebra **(F–J)**. Violin graphs showing distribution of values for volume of trabecular thickness **(A, F)**, connectivity density of trabeculae **(B, G)**, and mineralization of trabecular bone **(C, H)**. WT females in light blue, KO females in light red, WT males in dark blue, and KO males in dark red. Thick horizontal line marks median, red circle marks position of mean. Median specimens per group were chosen for representative images in **(D, E, I, J)**. Pseudocolors correspond to mid-range (mm) of the trabecular thickness **(D, I)** and their mineralization **(E, J)** in attenuation units (AU). Representative images are ordered the same way as groups in the graphs. Bar = 0.5 mm. Symbols for p-values of *post-hoc* multiple comparison test (provided after significant Kruskal-Wallis test) are as follows: + for p < 0.10; * for p < 0.05; ** for p < 0.01.

**Figure 4:**
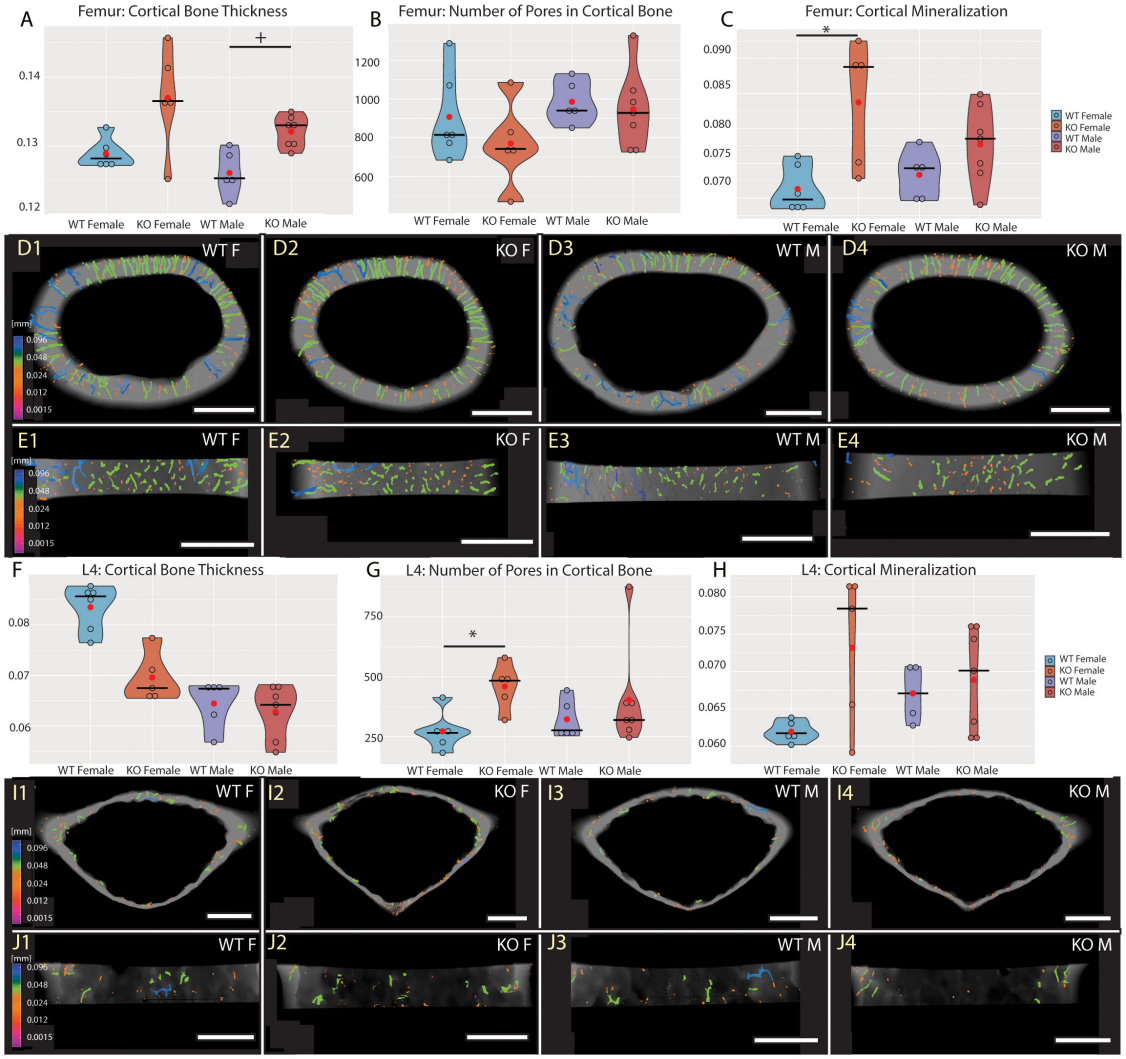
Cortical bone of central femur **(A–E)** and L4 vertebra **(F–J)**. Violin graphs showing distribution of values for volume of cortical bone thickness **(A, F)**, number of pores **(B, G)**, and cortical mineralization **(C, H)**. WT females in light blue, KO females in light red, WT males in dark blue, and KO males in dark red. Thick horizontal line marks median, red circle marks position of mean. Median specimens per group were chosen for representative images in **(D, E, I, J)**. Pseudocolors correspond to ESDv (Volume-equivalent sphere diameter, mm) of pores in transversal **(D, I)** and medial **(E, J)** section. Representative images are ordered the same way as groups in the graphs. Bar = 0.5 mm. Symbols for p-values of *post-hoc* multiple comparison test (provided after significant Kruskal-Wallis test) are as follows: + for p < 0.10; * for p < 0.05.

To investigate the role of Fgf20 in mineral homeostasis in adult bones, we have proceeded with detailed high-resolution microCT analysis and quantification of bone mineralization and ultrastructure parameters, such as thickness of trabecular and cortical bone, connectivity density of trabecular bone, pore quantity in cortical bone and mineral level in both trabecular and cortical bones. For detailed analysis, we selected a femur as representative of the long bone and a fourth lumbar vertebra (L4) for the axial skeleton (27). The ultrastructure of trabecular bone in the femur and the vertebra was significantly affected in both males and females (**Table 2**, **Figure 3**). The thickness of trabecular bone was significantly reduced in Fgf20 KO males compared to WT controls in both femur and L4 (**Figures 3A, D1–D4, F, I1–I4**). The connectivity of the femoral trabecular bone showed only a non-significant trend that was specifically visible in the Fgf20 KO males (**Figure 3B**). However, in the vertebra, connectivity of trabecular bone was significantly increased in Fgf20 KO females (**Figure 3G**). In both investigated bones, we observed a mild trend toward increased trabecular bone mineralization, particularly in Fgf20 KO female femur (**Figures 3C, E1–E4, H, J1–J4**). Contrary to the trabecular area, the cortical bone thickness in the femur was mildly increased specifically in Fgf20 KO males (**Table 2**, **Figures 4A, D1–D4, E1–E4**); however, the number of pores was unaffected (**Figure 4B**). The mineralization of femoral cortical bone increased in a similar pattern to that of femoral trabecular bone but with higher significance (**Figure 4C**), confirming the general role of Fgf20 in bone mineral deposition. There was only a non-significant trend toward a reduction of the cortical bone thickness in the lumbar vertebra in Fgf20 KO (**Figures 4F, I1–I4, J1–J4**). Nonetheless, number of pores increased significantly in the L4 of Fgf20 KO females (**Figures 4G, I1–I4, J1–J4**). The mineralization status of Fgf20 KO lumbar cortical bones was consistent with previous measurements in lumbar trabecular bone (**Figure 4H**).

**Table 2:** Analysis of bone structure and mineralization.

Area	Variable	Bone	H (3, N = 23)	p	Multiple Comparison	
					Females: WT/KO	Males: WT/KO
Trabecular	Thickness	Femur	11.74	0.01	1.00	**0.01**
		L4	15.64	0.00	**0.06**	**0.02**
	Connectivity Density	Femur	3.29	0.35	N/A	N/A
		L4	7.84	0.05	**0.04**	1.00
	Mineralization	Femur	8.01	0.05	**0.09**	1.00
		L4	5.21	0.16	N/A	N/A
Cortical	Thickness	Femur	10.92	0.01	0.17	**0.10**
		L4	14.31	0.00	0.32	1.00
	Number of Pores	Femur	3.51	0.32	N/A	N/A
		L4	8.41	0.04	**0.03**	1.00
	Cortical Mineralization	Femur	8.54	0.04	**0.05**	1.00
		L4	6.01	0.11	N/A	N/A

Results of the Kruskal-Wallis non-parametric test with a post-hoc multiple comparison test was used to detect the presence of significant differences among the four groups, i.e. WT females, KO females, WT males, and KO males. Only relevant results comparing WT and KO mice in each of the sexes separately are mentioned. Significant results (p < 0.05) in red, near-significant results (p < 0.1) in blue.

To conclude, in the Fgf20 KO mice, we spotted similar morphological trends in the trabecular and cortical bones, particularly prominent in the trabecular area. Interestingly, bone thickness tended to reduce in all investigated bones except for the femoral cortical bone, where it mildly increased.

### Fgf20 KO influences bone histoanatomy and osteoclast distribution

**Figure 5:**
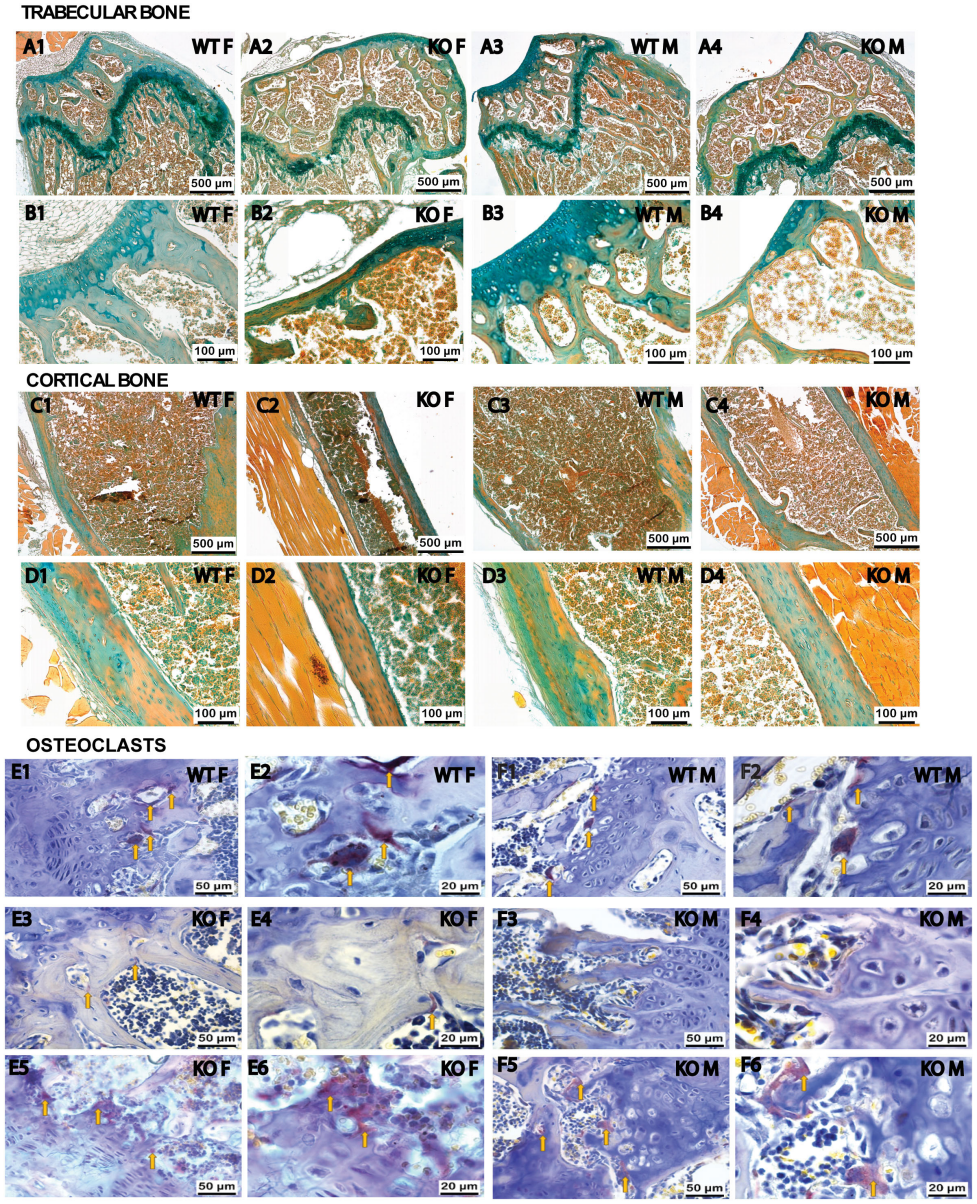
Histological staining of femur sections. Differences in the morphological organization of articular cartilage, growth plate, and bone tissue mineralization were revealed through Alcian blue and Alizarin red tissue-specific staining **(A1–D4)** on serial sections of femurs in WT **(A1–D1; A3–D3)** and Fgf20 KO **(A2–D2; A4–D4)** mouse groups. Alcian blue staining specifically targets decalcified, hyaline cartilage, whereas Alizarin red staining highlights calcified tissue. The growth plate of Fgf20 KO mice was disorganized and bone trabeculae were thinner **(A2, A4)** compared WT mice **(A1, A3)**. High magnification **(B1–B4)** reveals enhanced bone tissue formation and disruption of Fgf20 KO femur cartilage. Cortical bone mineralization was higher in Fgf20 KO mice **(C1–C4)**. The periosteum was thinned and visualized without a layered structure **(D1–D4)**. TRAP staining **(E1–F6)** was used to detect the distribution and morpho-functional features of osteoclasts (indicated by yellow arrows) in WT **(E1–F2)** and Fgf20 KO mice **(E3–F6)**. In Fgf20 KO animals, osteoclasts were less abundant in the central zone of the growth plate **(E3, F3)** than at the periphery **(E5, F5)**, where they were numerous and exhibited a degenerative morphology. Intense ossification processes were observed in the central part of the growth plate **(E4, F4)**, particularly pronounced in females **(E4)**. Osteoclasts in the KO group were vacuolated and clustered near the hyaline cartilage, disrupting its structure **(E6, F6)**, with higher activity in females **(E6)** than males **(F6)**. Scale bar shown (500, 100, 50 and 20 µm).

To comprehensively evaluate the physiological status of the bone, we conducted an in-depth histological examination. This analysis histologically validated the articular cartilage changes in Fgf20 KO mice, revealing thinning and replacement by bone tissue. On sections stained with Alcian blue and Alizarin red, the areas of mineralization in the articular cartilage were clearly visible (**Figures 5A1–B4**). Bone trabeculae were thinner in Fgf20 KO mice compared to WT mice (**Figures 5A1–A4**). Differences were also found in the morphological structure of the growth plate. The growth plate of Fgf20 KO mice was severely disorganized and hypertrophic chondrocytes were not observed as in WT mouse bones (**Figures 5A1–A4**).

Further histological analysis revealed that the increased ossifying matrix was thinner around the growth plate cartilage, and chondrocyte clusters tended to be directly exposed to the medullary cavity (**Figures 5A1–A4**). Alcian blue and Alizarin red staining showed that the thickness of growth plate cartilage was significantly reduced, and trabecular bone in the second ossification zone was notably lost in the Fgf20 KO group relative to the WT group. In addition, a marked accumulation of mineralized osteoids was seen in trabecular bone and secondary spongiosa (**Figures 5A1–A4**).

The structure of the cortical bone differed in Fgf20 KO mice from the group of WT mice. In bones from Fgf20 KO mice, cortical bone mineralization was higher than in WT mice; there were also areas of completely unmineralized cortical bone (**Figures 5C1–D4**). The periosteum was thinned and visualized without a layered structure (**Figures 5D1–D4**). These results suggest that Fgf20 KO mice enhanced bone tissue formation by promoting osteodifferentiation and extracellular matrix mineralization. Comparable observations were noted using Masson’s Trichrome staining (see **Supplementary Figures S2**, **S3** in the **Supplementary Material**).

Bone homeostasis is governed by the coordinated activities of osteoblasts and osteoclasts. Osteoclast numbers in the distal femur of mice were assessed through TRAP staining. In Fgf20 KO animals, the number of osteoclasts was lesser in the central zone of the growth plate (**Figures 5E3, F3**) compared to the periphery part (**Figures 5E5, F5**), where these cells were present and exhibited a spindle-shaped, degenerative morphology. This pattern was observed in both sexes. In the WT animal group, osteoclasts were distributed throughout the entire growth plate (**Figures 5E1, F1**). However, in Fgf20 KO animals, osteoclasts were vacuolated and clustered directly adjacent to the hyaline cartilage of the growth plate, leading to the disruption of normal cartilage structure with the absence of a hypertrophy zone and subsequent calcification (**Figures 5E6, F6**). This osteoclast activity was significantly higher in females (**Figure 5E6**) than in males (**Figure 5F6**). In the central part of the growth plate, intense ossification processes were observed (**Figures 5E4, F4**). The severity of growth cartilage ossification in females (**Figure 5E4**) was higher than in males (**Figure 5F4**) in the Fgf20 KO animal group. Significant cytoplasmic and border vacuolization (**Figures 5E6, F6**) of osteoclasts indicated intense processes of destruction and resorption of osteogenic components in the growth plate area. These osteoclasts stained by TRAP appeared lighter and larger than normal (**Figures 5E2, F2**). This heightened activity and localized, uneven, concentration of osteoclasts, along with areas without osteoclasts or zero functional activity in the growth plate, disrupt the normal remodeling of bone tissue, mediate bone loss in pathological conditions, and lead to pathological bone deformation.

### Fgf20 is involved in molar morphogenesis and root morphology

**Figure 6:**
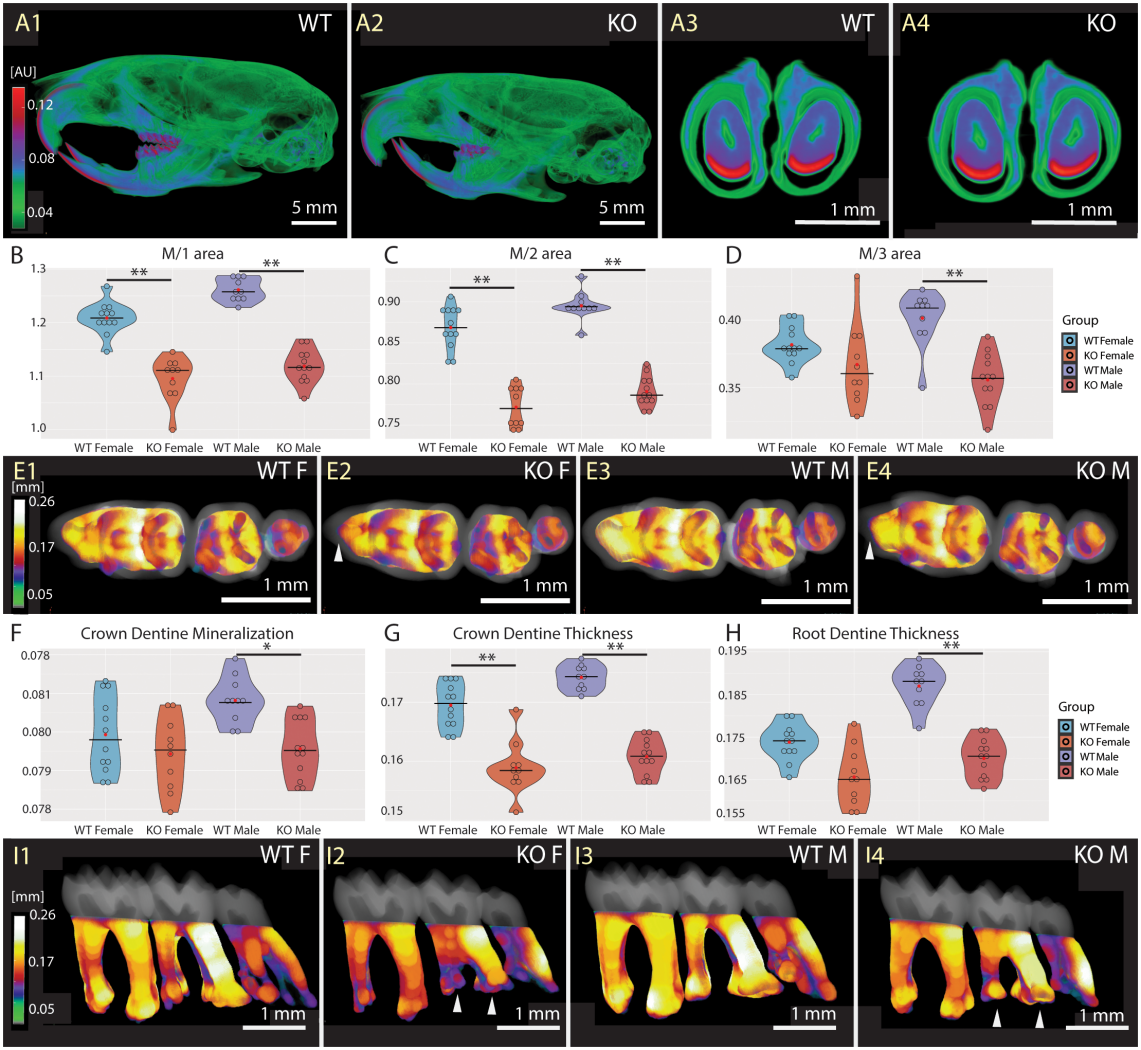
Mineralization and morphology of dentition. Comparison of WT **(A1, A3)** and Fgf20 KO **(A2, A4)** with respect to mineralization in dentition. The whole dentition in the context skull from the left side **(A1, A2)**, and sagittal section of lower incisors **(A3, A4)**. Pseudocolors correspond to mineralization **(A)** in attenuation units (AU). No striking differences were observed in that matter. Violin graphs showing distribution of values for area of the first **(B)**, second **(C)** and third **(D)** molar, crown dentine mineralization **(F)** and thickness **(G)**, and root dentine thickness **(H)**. WT females in light blue, KO females in light red, WT males in dark blue, and KO males in dark red. Thick horizontal line marks median, red circle marks position of mean. Pseudocolors correspond to mid-range (mm) of the dentine thickness in crown **(E1–E4)** and root **(I1–I4)**. Indicated by a brighter pseudocolor, the crown dentine is apparently thicker in WTs **(E1, E3)** in comparison to Fgf20 KOs **(E2, E4)**. Notably, the anteroconid of M/1s, pointed out by a white arrowhead, is less prominent in KOs **(E2, E4)**. In roots, thicker dentine is observed again in WTs **(I1, I3)** compared to Fgf20 KOs **(I2, I4)**, except for distal M/3s. Taurodontism is visible in the M/2 of Fgf20 KOs, highlighted with white arrowheads, and M/3 roots are less differentiated **(I2, I4)**. Bar = 5.0 mm for **(A1, A2)**, and 1.0 mm for **(A3, A4)**, **(E1–E4)**, **(I1–I4)**. Symbols for p-values of *post-hoc* multiple comparison test (provided after significant Kruskal-Wallis test) are as follows: * for p < 0.05; ** for p < 0.01.

Since Fgf20 is expressed during tooth development and ameloblast differentiation (19, 28), we proceeded with a detailed analysis of tooth morphology and mineralization status. The high-resolution microCT imaging and further analysis showed no morphological differences in Fgf20 incisors, nor any changes in incisor mineral density in enamel and dentin (**Figures 6A3, A4**). The mineralization status of craniofacial and skull bones was not affected by Fgf20 ablation as well (**Figures 6A1, A2**). We assume that the possible role of Fgf20 in mineral homeostasis in cranial bones and incisors likely is compensated by other Fgf signalling molecules.

The morphology of molars in Fgf20 KO mice is altered, featuring reduced size (**Figures 6B–D**), less diverse crowns and roots (**Figures 6E1–E4, I1–I4**) and thinner dentine (**Figures 6E1–E4, G, H, I1–I4**). The less differentiated crown pattern is notably evident in the reduced prominence of the anteroconid of M/1s (**Figures 6E2, E4**). The root morphology, especially in M/2s, may resemble taurodontia in ceratin animals (**Figures 6I2, I4**). Males exhibit reduced mineralization of dentin in the crown (**Figure 6F**). The difference in dentine thickness in the crown is observed in both sexes (**Figure 6G**), while in the roots is, again, significantly reduced only in males (**Figure 6H**). However, mineralization and developmental dynamics of the molar tooth row do not appear to be affected. See **Supplementary Tables S3**-**5** in the **Supplementary Material** for detailed results.

To conclude, the phenotype of Fgf20 KO mutation is more prominent in molars of males, where significant differences were found not only in dentine thickness, but also in dentine mineralization of the crown.

## Discussion

### Fgf20 role in digit identity

The most notable phenotype in Fgf20 KO mice was an alteration in digit number and morphology (**Figures 1G, H**). The morphogenesis of digits is mediated by the apical ectodermal ridge (AER), an epithelial structure secreting multiple Fgf ligands, including Fgf20 (15). The secretion results in a positive feedback circuit and reciprocally controls the zone of polarizing activity (ZPA). ZPA is a major regulatory source of Sonic hedgehog signalling, affecting the digit number and morphology (29, 30). Abnormalities in AER structures have been shown to lead to the polydactyly phenotype before (31, 32). Furthermore, alteration in ZPA function can advance to the digit loss phenotype as an outcome of a reduction in Sonic hedgehog (Shh) distribution (33). A digit loss phenotype was observed in a mouse model with conditionally inactivated fibroblast growth factor receptor 1 (FgfR1) and authors suggested that Fgfr1 regulation of Shh expression was involved (34). Nonetheless, Fgf20 was reported not to interact with Shh in hair placodes (18), implying a different possible regulatory network involved.

### Role of Fgf20 in bone homeostasis

It has been suggested that Fgf20 might have redundant roles with other members of the Fgf9 subfamily (15, 22, 35), specifically Fgf9 and Fgf16. However, the differential temporospatial regulation of expression might provide a unique mechanism by which the Fgf signal is translated into physiological function. Fgf20 is a downstream target of the Wnt signalling network (36), as evidenced by its expression in patients with dysregulated Wnt signalling pathway, particularly in myelodysplastic syndromes (37).

Based on expression data, the source of the Fgf20 signal can be the stromal population of the hematopoietic stem cell niche (37), which can influence the subpopulation of osteoclasts in the area, since the receptor binding specificity is similar for all members of the Fgf9 subfamily (**Figures 2A, B**) (3). Differential levels of Fgf expression are anticipated during dynamic cellular processes. For instance, Fgf9 and Fgf16 have been reported to reach maximal expression in the late response after fracture (38). This suggests potential changes in expressions during ongoing chondrogenesis and osteogenesis.

Results from the Fgf9 and Fgf20 stimulation in MC3T3-E1 cells suggest that Fgf9 and Fgf20 might have regulatory effects in osteoblast progenitor to mature osteoblastic differentiation (**Figure 2C**). Fgf9 possibly exhibits a prolonged effect due to its increased protein stability (39), leading to a higher level of attenuation in the medium to avoid excessive impact or over-effect. Previously, it was described, that overexpression of Fgf9 in mouse chondrocytes leads to dwarfism phenotypes (40). On the other hand, Fgf20, being less stable and short-lived, is suitable for specific rapid effects, providing a transient boost. Moreover, Fgf20 demonstrates a higher affinity to the receptor, suggesting greater potency in selected progenitors. Variability in Fgf stability could be a fundamental factor regulating Fgf activity in developmental processes co-regulated by multiple Fgf ligands (39). Disbalance of this regulatory system may then lead to a developmental malformation.

The bone homeostasis phenotype was not reported in Fgf20 KO yet (22); however, Fgf20 was suggested as a gene involved in mineral homeostasis by collaborative cross analysis (21). Such evidence can also be supported by the phenotype of Fgf9 heterozygote mice (25), which mimics the Fgf20 phenotype described by us (**Figures 3C, E1–E4, H, J1–J4**, **Figures 4C, H**). Additionally, Fgf9, recognized for its impact on lung size through mesenchymal proliferation (41), plays crucial roles in development and repair of the skeletal system (42). In Fgf9 KO mice, a significant decrease in trabecular bone and bone formation in the femur was described, reflecting our result, along with a notable decrease in cortical thickness. However, female Fgf9 KO mice exhibit no changes in bone mass (43), which is inconsistent with our findings.

The histological examination of the femur in Fgf20 knockout mice is partially consistent with observations indicating that the absence of Fgf9 results in a reduction in osteoclast cell populations in the perichondrium and primary spongiosa during bone development. In Fgf20, the cells exhibited disorganized pattern (**Figures 5E3–E6, F3–F6**) rather than a reduction in number. Additionally, the analysis of Fgf9 KO limbs indicated that the loss of Fgf9 function leads to reduced chondrocyte proliferation, delayed initiation of chondrocyte hypertrophy and abnormal formation of mineralized bone (44), mirroring our findings (**Figures 5A1–B4**). This suggests that Fgf20 may be involved in regulating hypertrophic chondrocyte differentiation and the recruitment of osteoclasts to the growth plate.

### Fgf 20 as a part of the Eda and Wnt pathways

Fgf20 has been described as the downstream target of Eda signalling in developing tooth germs, and it has been proposed as an important mediator of Eda signalling in the tooth morphogenesis process (19). Eda ablation affects tooth morphogenesis, specifically by reducing tooth morphology. Despite tooth dysmorphology in Eda-deficient mice, the complex enamel structure and mineral density were not altered (45). In our data, changes in the density of the dental enamel of Fgf20 KO mice were not detected either (**Figures 6A1–A4**). We observed notable alterations in teeth morphology, featuring smaller molars (**Figures 6B–D**) with more uniform crown pattern (**Figures 6E1–E4**) reminiscent of the Eda phenotype (19). Additionally, Fgf20 KO males exhibited decreased dentin mineralization in the crown and reduced dentin thickness in the roots (**Figures 6F, H**). Moreover, crown dentine thickness was altered in both sexes (**Figure 6G**). The root dysmorphology resembling taurodontism were noted, particularly in M/2s, in selected KO mice (**Figures 6I2, I4**). Misshapened molar crown and root taurodontism were similarly reported in Wnt10a null mice, along with supernumerary fourth molars, which were not present in our case (46).

Interestingly, we observed similar skeletal malformations in the distal portion of the tail in Fgf20 KO mice (**Figure 1B**) as were reported in Eda pathway deficient mouse lines (47). Since Fgf20 is a downstream effector of the Eda pathway (18, 19), this data points to nonredundant roles of Fgf20 not only in bone homeostasis but also during skeletal development.

In the context of broader developmental networks, the absence of Fgf20 may affect the harmonious regulation of different signaling pathways, leading to faulty development of molars and the axial skeleton, resembling the phenotypes observed in Wnt and Eda ablation.

## Conclusion

We described skeletal malformations in mice lacking Fgf20 in the lumbar and caudal parts of the spine and in the digits at varying degrees of severity. Furthermore, quantitative computed tomography data show changes in the composition of trabeculae and cortical bone in long bones and vertebrae, particularly with increased mineral density. The bone physiological status was also evaluated by histological examination.

The study demonstrated effects on selected osteoblast progenitors to mature osteoblastic differentiation. Moreover, it indicated a diffuse pattern of Fgf20 signal through the long bone tissue, which was specifically mapped mainly into chondrocytes. Uneven concentration of osteoclasts and their activity through the femur tissue was revealed. Decrease in mineralization and dentin thickness were also observed in molars, along with abnormal morphology. Molars exhibited reduction in size, less differentiated crown patterning and root taurodontism. In contrast, enamel mineral density was unaltered.

Taken together, our data suggest that preservation of normal bone homeostasis, mineralization, skeletal growth and dental development rely on Fgf signalling with Fgf20 being one of the substantial molecules involved in bone differentiation regulatory network.

## Materials and methods

We collected data from 12 Fgf20 knockout mice, comprising 7 males and 5 females, and 11 wild-type control mice, consisting of 5 males and 6 females, all at the age of 13 weeks. Knockout mouse models were generated under the umbrella of IMPC (48) and so are distributed.

Mice are housed in individually ventilated cages (Tecniplast, Italy) with free access to water and rodent food (Altromin, Germany), on Safe Select Fine bedding (Velaz, Czech Republic) in a 12/12 light-dark cycle, maintaining a constant temperature between 20 and 24°C and humidity between 45 and 70%. The environment is enriched with either paper nests or red plastic igloos (Velaz, Czech Republic). The animals are cared for daily by qualified staff in accordance with current welfare rules and under veterinary supervision.

This study was carried out in a strict accordance with the Czech national laws and guidelines on the use of experimental animals and protection of animals against cruelty (Animal Welfare Act No. 246/1992 Coll.). The protocol was approved by the Committee on the Ethics of Institute of Molecular Genetics of the Czech Academy of Sciences, and the Departmental Expert Committee for the Approval of Projects of Experiments on Animals of the Academy of Sciences of the Czech Republic (AVCR 7341–2021 SOV II valid until 31.12.2026).

The studies were conducted as per the CCP Standard Operating Procedures (SOPs). Use of live animals was inevitable to accomplish the purpose of the study. The study was designed to use the minimum number of animals to meet the scientific objectives, the goals of the project and in consideration of applicable regulatory requirements.

### Mouse model

The Fgf20 KO mouse model was generated on a C57BL/6N background (Charles River Laboratories, USA) by targeting exon 2 (ENSMUSE00000582475). A frame-shifting deletion was generated using CRISPR/Cas9 technology. The guide RNAs (gRNAs) of highest score and specificity were designed using Crispor (49). The following guides were selected: gRNA1: GGAAAGCTATATTTGTTGTA, and gRNA2: TGGGTACTTAGTAAGCCATA. The gRNAs (100ng/µl) were assembled into a ribonucleoprotein (RNP) complex with Cas9 protein (500ng/µl; 1081058, 1072532, Integrated DNA Technologies, USA), electroporated into 1-cell zygotes, and transferred into pseudopregnant foster mice (Crl: CD1(ICR), Charles River Laboratories, USA). Putative founders were analyzed by PCR and sequencing. A founder harboring a 1362 bp deletion, including exon 2, was chosen for subsequent breeding. Genotyping was performed by PCR with forward (F) 5′-CAAGTTCCTTACAAAAGCCTCA-3′ and reverse (R) 5’-TCCTCTGCGAGAACAATGTG primers. The F and R products are 1721 bp in wild-type animals and 359 bp in mutant animals. The selected founder was bred with the C57Bl/6NCrl wild-type to confirm germ-line transmission of the target deletion. We further bred heterozygotes to expand the colony and generate homozygotes, which were the subjects of this study. Established strain will be available in the IMPC/EMMA repository in the CCP node as Fgf20^em1(IMPC)Ccpcz^.

For the whole-body imaging, all mice were anesthetized by intramuscular administration of 20% Zoletil-Xylazine and after the procedure, euthanized by cervical dislocation for further tissue collection. For dental enamel and bone microarchitecture analysis, skulls, femurs and fourth lumbar vertebrae were dissected. The fourth lumbar vertebra was selected as the morphological standard representing the axial skeleton. Femur and vertebral samples were fixed in paraformaldehyde (PFA) for 1–2 weeks and embodied in 2.5% agarose gel afterwards.

### Whole body skeleton analysis

Experimental animals were scanned *in-vivo* using SkyScan 1176 (Bruker, Belgium) at voxel size 35 μm, 0.5 mm aluminum filter (source voltage = 50 kV, source current = 246 µA, rotation step = 0.7°) with 180° rotation. Data were reconstructed using InstaRecon CBR Premium, version 2.0.4.0 (InstaRecon, USA) with the following settings: smoothing = 3, ring artefact correction = 4, beam hardening = 36%, with spread of intensities 0.0047 – 0.1230 AU. For 3D visualization and bone morphology evaluation, CTvox software, version 3.3.0.0 (Bruker, Belgium) was used.

### Bone microarchitecture analysis

Bone ultrastructure was imaged ex-vivo using the Skyscan 1272 micro-CT system (Bruker, Belgium). Used settings, stated by the manufacturer as the most suitable for skeletal analysis, were the following: pixel size 1,5 µm, energy filter 1 mm aluminum, 360° scanning, rotation step 0.21° with averaging = 2 applied. Reconstructions were performed in InstaRecon CBR Premium, version 2.0.4.0 (InstaRecon, USA) with set up for femurs: smoothing = 6, ring artefact correction = 8, beam hardening = 28%, intensity distribution = 0.0000 – 0.1100. Vertebrae were reconstructed within the same set of parameters except for the spread of intensities, which was set as follows: 0.0000 – 0.125000.

Subsequently, DataViewer 1.5.2 (Bruker, Belgium) was used for data reorientation and CT Analyzer 1.16.4.1 (Bruker, Belgium) was used for volume of interest (VOI) definition in the central diaphysis for cortical bone and distal metaphysis for trabecular bone of femur and caudal part of vertebral body. Then, with the same software, the detailed bone analysis was performed in specified VOIs according to our standardized procedure (50). Cortical and trabecular bone thickness, connectivity density of trabeculae, and number of pores in cortical bone were selected as the best describing parameters of spatial bone structure. Bone mineralization was established based on the strength of the signal in Attenuation Units (AU) inside VOIs. Statistical analysis was processed with the help of Statistica 13 (Statsoft, USA). Kruskall-Wallis non-parametric test was used to detect presence of significant differences among four groups, i.e. WT females, KO females, WT males, and KO males. If significant, *post-hoc* multiple comparison test was applied to find significance between particular groups. The violin plots were produced in R, version 4.2.2 (51) within R Studio 2022.07.2 (52) application and with ggplot2 3.4.0 library (53).

### Molar analysis

After euthanizing the mice using cervical dislocation, the skulls were carefully extracted, cleared of soft tissue with a scalpel and immersed in a 5% H_2_O_2_ solution for 5 days. Following dehydration, the skulls were scanned using a SkyScan 1176 (Bruker, Belgium) at a resolution of 9 µm with a 0.5 mm aluminum filter, employing a full rotation scan with a 0.33° step. Data reconstruction was performed using NRecon 1.7.3.1 (Bruker, Belgium) with InstaRecon 2.0.4.0 (InstaRecon, USA) add-on, utilizing a range of intensities from 0.005 to 0.200 AU, smoothing = 3, ring artifact correction = 4, and beam hardening correction = 36%.

Following the reconstruction process, the DataViewer 1.5.2 (Bruker, Belgium) was used to measure the maximum length and width of all lower molars. Subsequently, the square area of all molars was computed, along with the elongation ratio of M/1 determined by its length and width. Additionally, the ratio between the square area of M/2 and M/1 was analyzed to assess changes in developmental dynamics in the tooth row.

Subsequently, the molar area was manually segmented using CT Analyzer 1.16.4.1 (Bruker, Belgium). After reorientation to the occlusal plane in DataViewer, semi-automatic segmentation of molars from the mandibular bone was performed in CT Analyzer, utilizing a task list. Initially, molar enamel was segmented and analyzed with Otsu’s method segmentation in CT Analyzer to prevent artifacts arising from varying levels of mineralization. Following this, dentine was manually divided into crown and root areas, each analyzed separately in CT Analyzer (54).

Considering the morphometric results mentioned above, the following parameters were selected as the most suitable for a detailed description of molar structure: mineralization of all three parts in AU, mean thickness of all three parts, surface-to-volume ratio of crown and dentine pulp to illustrate spatial diversity, and the relative volume of the pulp to the total crown or root volume. This selection aims to clarify whether changes in pulp size correspond to alterations in the mineralized portions of the molars.

Statistical analysis followed the same procedure as in the bone microarchitecture analysis. Primary data are available in **Supplementary Table S3**, means and standard deviation per each group in **Supplementary Table S4** and results of the Kruskal-Wallis non-parametric test with a *post-hoc* multiple comparison test, detecting variance among the four groups, i.e. WT females, KO females, WT males, and KO males, in **Supplementary Table S5** in the **Supplementary Material**.

### RNA *in situ* hybridization

Femurs from 5-week-old wild-type mice were fixed in 4% PFA, decalcified in Morse’s solution, embedded in paraffin, and sectioned at 10 μm. Hybridization on sections was performed overnight at 70°C with a DIG-labeled Fgf20 probe in hybridization buffer (5X saline-sodium citrate, pH 7.0, 50% formamide, 0.1% Tween-20, Denhardt´s solution, heparin (50 μg/ml), tRNA (50 μg/ml), and salmon sperm DNA (50 μg/ml)). The negative control sections were hybridized with the Fgf20 sense probe (complementary to the sequence of the Fgf20 probe). The digoxygenin-labeled RNA probes (DIG RNA labelling kit, Roche, France) were generated by *in vitro* transcription from a PCR-amplified fragment of murine Fgf20. The sequence of the Fgf20 antisense probe is shown in **Supplementary Table S2**. An anti-DIG antibody conjugated to alkaline phosphatase and BM purple alkaline phosphatase substrate precipitating solution were used for staining. Stained sections were post-fixed with 4% PFA and imaged using the Axio Imager 2 (Zeiss, Germany).

### Fgf9 subfamily members expression analysis

The data was retrieved from Zhong et al. (24), available in GEO under accession code GSE145477. We ran the data through a custom pipeline with per-cell quality control metric calculated with the function perCellQCMetrics() from the scater package (55), and cells with over 5% mitochondrial expression were discarded. Then, we removed ribosomal genes, as described in the KEGG ribosome set (56), available as one of the mouse-curated (C2) gene sets from the Molecular signatures database (57), using the function msigdbr from the msigdbr package (58). Library size factors and logarithms of the read counts were calculated with functions from the package SingleCellExperiment (59). The variance of the log-counts was modeled with modelGeneVar() function from the scran package (60), and the genes with positive biological variance and FDR < 0.05 were used as a subset to calculate the Principal Components with the function runPCA() from the scater package (55), followed by t-distributed Stochastic Neighbor Embedding (t-SNE) calculation with the function runTSNE(). The cell annotation was taken from Zhong et al. metadata file (24), available at the single cell Broad Institute website.

### FGF9 and FGF20 stimulation in MC3T3-E1 cells

MC3T3-E1 cells were seeded in T75 tissue culture flasks using an expansion medium composed of Alpha Minimum Essential Medium (α-MEM) containing 10% fetal calf serum (FCS), 1% penicillin/streptomycin, and 1% glutamine. Cell cultures were maintained at 37 ˚C in a humidified atmosphere of 95% air and 5% CO2, with media changes every 2–3 days. Cell proliferation was determined by cell counting using a Coulter counter (Beckman Coulter, USA). MC3T3 cells were plated at 1 × 10^5 cells/well in six-well plates. On day 0, the culture media were replaced with α-MEM supplemented with 0.1% (v/v) FCS, 50 μg/ml gentamicin, and 30 ng/ml FGF-9 (7399-F9, Bio-Techne R&D Systems, UK) or Fgf-20 (CSB-EP008626MO, Cusabio, USA),. The differentiation culture was conducted with and without the growth factors for 6 hours. Each test condition was performed in triplicate. The effects of Fgf9 and Fgf20 on the mRNA expression levels of *Col1a1, Sost, Alpl, Sparc*, and *Osteocalcin* were examined. See the primers in **Supplementary Table S1** in the **Supplementary Material**.

Total RNA was extracted from MC3T3-E1 cells using a Qiagen RNeasy Mini kit (Qiagen, UK) following the manufacturer’s recommendations. The RNA samples (0.5 µg) were reverse-transcribed into cDNA using the Reverse Transcription System (PR-A3500, Promega, USA) according to the manufacturer’s instructions. RT-qPCR was carried out in a LightCycler 480 cycler (Roche, France). The qPCR replicates were averaged, and to get delta Cq values, the reference gene Rpl19 was subtracted from the gene of interest. The mean of the control samples was subtracted from each sample to get delta-delta Cq. These values were recalculated to relative values when the mean of the control samples was set to 1. The statistical analysis was performed in R, version 4.2.2 (51), using the lmer package (61) and a mixed linear model with a qPCR date as a random effect, followed by Dunnett’s post-test.

### Histological analysis

Samples of bones for histopathology were processed manually under RNA-free conditions according to internal SOP, embedded, and sectioned by a Leica RM2255 rotary microtome (Leica Biosystems Nussloch GmbH, Germany) with a thickness of 4.5 µm. Histological sections were stained by Masson’s Trichrome using the Ventana BenchMark automatic staining machine (Roche, France) with the Trichrome staining kit (LOT: K07708), which is a commonly used method in bone histology and allows tissue identification by different coloring as well as by morphological identification. For simultaneously visualizing cartilage and mineralized tissues, histological sections were manually stained with Alcian blue 8GX (LOT: SLBR0633V, Merck, Germany) and Alizarin red S (LOT: MKCC2711, Merck, Germany) according to internal SOP. Leukocyte acid phosphatase (TRAP) kit (LOT: SLCJ3762, Merck, Germany) was used to detect TRAP granules as cytochemical markers of osteoclasts. All slides were coverslipped with Histolab Pertex Mounting Medium (Histolab Products AB, Sweden) by the automatic coverslipper machine Leica CV5030 (Leica Biosystems Nussloch GmbH, Germany).

## Data availability statement

The raw data supporting the conclusions of this article will be made available by the authors, without undue reservation.

## Ethics statement

The animal study was approved by Animal welfare and ethical committee of Institute of molecular genetics Czech Academy of Sciences. The study was conducted in accordance with the local legislation and institutional requirements ethical approval number: 7341-2021 SOVII.

## Author contributions

SD: Formal analysis, Investigation, Methodology, Writing – original draft. FS: Conceptualization, Data curation, Investigation, Methodology, Software, Writing – original draft, Writing – review & editing. CT: Investigation, Methodology, Writing – review & editing. BO: Investigation, Methodology, Writing – review & editing. MP: Investigation, Writing – review & editing, Methodology. OF: Investigation, Writing – review & editing. PN: Investigation, Writing – review & editing, Methodology, Resources. GA: Investigation, Methodology, Writing – review & editing. RS: Conceptualization, Funding acquisition, Resources, Supervision, Writing – review & editing. JP: Conceptualization, Data curation, Formal analysis, Funding acquisition, Investigation, Supervision, Writing – review & editing, Project administration, Resources.
