# Supplementary material for: Skeletal dysmorphology and mineralization defects in Fgf20 KO mice
Source: Front Endocrinol (Lausanne). 2024 Jul 26;15:1286365. doi: 10.3389/fendo.2024.1286365 (PMC11310068; doi:10.3389/fendo.2024.1286365)
Supplement: Supplementary file 1 [file DataSheet_1.docx]

Supplementary Material

**Skeletal dysmorphology and mineralization defects in Fgf20 KO mice**

Sylvie Dlugosova^1^, Frantisek Spoutil^1^, Carlos Eduardo Madureira Trufen^1^, Betul Melike Ogan^2^, Olha Fedosieieva^1^, Michaela Prochazkova^2^, Petr Nickl^1^, Goretti Aranaz Novaliches^2^, Radislav Sedlacek^1,2^, Jan Prochazka^1,2*^

1. Czech Centre for Phenogenomics, Institute of Molecular Genetics of the Czech Academy of Sciences, v.v.i., 252 50 Vestec, Czech Republic
2. Laboratory of Transgenic Models of Diseases, Institute of Molecular Genetics of the Czech Academy of Sciences, v.v.i., 252 50 Vestec, Czech Republic

*corresponding author: [jan.prochazka@img.cas.cz](mailto:jan.prochazka@img.cas.cz)

**Table S1:** Primers for qPCR.

|  | Forward | Reverse |
| --- | --- | --- |
| *Rpl19 (RG)* | AAGCCTGTGACTGTCCATTC | GATCCTCATCCTTCTCATCCAG |
| *Col1a1* | CCTCAGGGTATTGCTGGACAAC | CAGAAGGACCTTGTTTGCCAGG |
| *Sost* | AGGAATGATGCCACAGAGGTC | TTTGGCGTCATAGGGATGGT |
| *Alpl* | GCACCTGCCTTACCAACTCT | TCAAGGTCTCTTGGGCTTGC |
| *Sparc* | GTTGGCCCGAGACTTTGAGA | AGCTCAGTGTGGGACAGGTA |
| *Osteocalcin* | CGCTCTGTCTCTCTGACCTC | GCCGGAGTCTGTTCACTACCT |

**Table S2:** Fgf20 antisense probe sequence.

Ggactggtcagtatcagaggtgtggacagtggcctgtaccttgggatgaatgacaaaggagaactttatggatcagagaaattgacttctgaatgcatcttcagggaacaatttgaagagaactggtataatacctattcgtccaacatatataaacatggagacacgggtcgcaggtattttgtagcacttaacaaggatggaactccaagagatggtgccaggtccaaaagacatcaaaagtttacccactttttaccaagaccagtagacccagaaagagttccagaattatacaaagacctactgatgtacacttgatgaatctagagccattgtttaaaaatcacagttcctgctgttaaataacaccgaagaagacgttcaggatattacgggagtctgcttttcactgaaagactctatttgggaagaaaattgagagtaaggaattaacttgaagcaaagcaagatcattctccgtaagtggattgtagttccttagacacgttgtttcagtcttaccagtagactgacgatgctgaaatcagttcatctgcggataatgtgaaccttgctgctgacgccgcatgtctctggataatgtttacttggacagttatcttaaaaatagatacttcatgttgaagaagtggattgagatgacataattactgcctcataaattctgaggaccttgtagaaaggttagaacgttatacataaaacaaaaatcaaaatactagatgactttgatctacaaaccacaccacactgag

**Table S3:** Primary data of utilized variables along with basic metrics of molars.

| **Strain** | **Sex** | **No.** | **Side** | **Length of M/1** | **Width of M/1** | | | | **Length of M/2** | | **Width of M/2** | | | | **Length of M/3** | | | **Width of M/3** | |  |
| --- | --- | --- | --- | --- | --- | --- | --- | --- | --- | --- | --- | --- | --- | --- | --- | --- | --- | --- | --- | --- |
| Fgf20 | Female | 64U-G4412 | Right | 1.286 | 0.777 | | | | 0.848 | | 0.891 | | | | 0.676 | | | 0.640 | |  |
| Fgf20 | Female | 64U-G4412 | Left | 1.367 | 0.816 | | | | 0.881 | | 0.905 | | | | 0.621 | | | 0.624 | |  |
| Fgf20 | Female | 64U-G8045 | Right | 1.303 | 0.835 | | | | 0.848 | | 0.884 | | | | 0.571 | | | 0.597 | |  |
| Fgf20 | Female | 64U-G8045 | Left | 1.312 | 0.849 | | | | 0.903 | | 0.878 | | | | 0.554 | | | 0.593 | |  |
| Fgf20 | Female | 64U-G8046 | Right | 1.283 | 0.831 | | | | 0.900 | | 0.872 | | | | 0.601 | | | 0.591 | |  |
| Fgf20 | Female | 64U-G8046 | Left | 1.281 | 0.835 | | | | 0.863 | | 0.861 | | | | 0.547 | | | 0.632 | |  |
| Fgf20 | Female | 64U-G8049 | Right | 1.316 | 0.851 | | | | 0.880 | | 0.900 | | | | 0.608 | | | 0.640 | |  |
| Fgf20 | Female | 64U-G8049 | Left | 1.318 | 0.869 | | | | 0.886 | | 0.909 | | | | 0.571 | | | 0.653 | |  |
| Fgf20 | Female | 64U-G8312 | Right | 1.322 | 0.853 | | | | 0.814 | | 0.916 | | | | 0.576 | | | 0.612 | |  |
| Fgf20 | Female | 64U-G8312 | Left | 1.301 | 0.851 | | | | 0.849 | | 0.885 | | | | 0.571 | | | 0.640 | |  |
| Fgf20 | Male | 64U-G4411 | Right | 1.315 | 0.832 | | | | 0.849 | | 0.901 | | | | 0.623 | | | 0.607 | |  |
| Fgf20 | Male | 64U-G4411 | Left | 1.352 | 0.858 | | | | 0.862 | | 0.891 | | | | 0.573 | | | 0.612 | |  |
| Fgf20 | Male | 64U-G8039 | Right | 1.273 | 0.831 | | | | 0.900 | | 0.883 | | | | 0.607 | | | 0.606 | |  |
| Fgf20 | Male | 64U-G8039 | Left | 1.316 | 0.865 | | | | 0.919 | | 0.872 | | | | 0.588 | | | 0.607 | |  |
| Fgf20 | Male | 64U-G8040 | Right | 1.270 | 0.865 | | | | 0.902 | | 0.865 | | | | 0.570 | | | 0.591 | |  |
| Fgf20 | Male | 64U-G8040 | Left | 1.316 | 0.848 | | | | 0.923 | | 0.885 | | | | 0.519 | | | 0.644 | |  |
| Fgf20 | Male | 64U-G8041 | Right | 1.336 | 0.854 | | | | 0.906 | | 0.867 | | | | 0.555 | | | 0.574 | |  |
| Fgf20 | Male | 64U-G8041 | Left | 1.335 | 0.876 | | | | 0.893 | | 0.903 | | | | 0.606 | | | 0.640 | |  |
| Fgf20 | Male | 64U-G8042 | Right | 1.318 | 0.845 | | | | 0.871 | | 0.904 | | | | 0.554 | | | 0.628 | |  |
| Fgf20 | Male | 64U-G8042 | Left | 1.302 | 0.836 | | | | 0.876 | | 0.889 | | | | 0.591 | | | 0.608 | |  |
| Fgf20 | Male | 64U-G8044 | Right | 1.355 | 0.832 | | | | 0.900 | | 0.868 | | | | 0.595 | | | 0.627 | |  |
| Fgf20 | Male | 64U-G8044 | Left | 1.350 | 0.827 | | | | 0.918 | | 0.898 | | | | 0.554 | | | 0.644 | |  |
| WT | Female | 82Z-10501 | Right | 1.417 | 0.831 | | | | 0.923 | | 0.918 | | | | 0.571 | | | 0.663 | |  |
| WT | Female | 82Z-10501 | Left | 1.436 | 0.883 | | | | 0.900 | | 0.917 | | | | 0.589 | | | 0.661 | |  |
| WT | Female | 82Z-10510 | Right | 1.436 | 0.851 | | | | 0.950 | | 0.934 | | | | 0.642 | | | 0.629 | |  |
| WT | Female | 82Z-10510 | Left | 1.410 | 0.851 | | | | 0.934 | | 0.936 | | | | 0.555 | | | 0.663 | |  |
| WT | Female | 82Z-10511 | Right | 1.315 | 0.871 | | | | 0.976 | | 0.884 | | | | 0.555 | | | 0.644 | |  |
| WT | Female | 82Z-10511 | Left | 1.402 | 0.859 | | | | 0.900 | | 0.921 | | | | 0.593 | | | 0.630 | |  |
| WT | Female | 82Z-10530 | Right | 1.367 | 0.875 | | | | 0.919 | | 0.936 | | | | 0.591 | | | 0.641 | |  |
| WT | Female | 82Z-10530 | Left | 1.421 | 0.865 | | | | 0.954 | | 0.935 | | | | 0.589 | | | 0.640 | |  |
| WT | Female | 82Z-10531 | Right | 1.402 | 0.868 | | | | 0.952 | | 0.934 | | | | 0.610 | | | 0.646 | |  |
| WT | Female | 82Z-10531 | Left | 1.437 | 0.844 | | | | 0.953 | | 0.901 | | | | 0.589 | | | 0.643 | |  |
| WT | Female | 82Z-10532 | Right | 1.420 | 0.865 | | | | 0.952 | | 0.952 | | | | 0.593 | | | 0.640 | |  |
| WT | Female | 82Z-10532 | Left | 1.372 | 0.876 | | | | 0.975 | | 0.914 | | | | 0.646 | | | 0.623 | |  |
| WT | Male | 82Z-10667 | Right | 1.436 | 0.869 | | | | 0.977 | | 0.953 | | | | 0.607 | | | 0.644 | |  |
| WT | Male | 82Z-10667 | Left | 1.402 | 0.889 | | | | 0.940 | | 0.952 | | | | 0.624 | | | 0.643 | |  |
| WT | Male | 82Z-10668 | Right | 1.426 | 0.871 | | | | 0.937 | | 0.953 | | | | 0.621 | | | 0.667 | |  |
| WT | Male | 82Z-10668 | Left | 1.423 | 0.896 | | | | 0.940 | | 0.957 | | | | 0.607 | | | 0.674 | |  |
| WT | Male | 82Z-10669 | Right | 1.455 | 0.862 | | | | 0.969 | | 0.936 | | | | 0.608 | | | 0.642 | |  |
| WT | Male | 82Z-10669 | Left | 1.449 | 0.889 | | | | 0.937 | | 0.952 | | | | 0.610 | | | 0.693 | |  |
| WT | Male | 82Z-10670 | Right | 1.442 | 0.891 | | | | 0.940 | | 0.952 | | | | 0.627 | | | 0.652 | |  |
| WT | Male | 82Z-10670 | Left | 1.420 | 0.888 | | | | 0.953 | | 0.934 | | | | 0.625 | | | 0.660 | |  |
| WT | Male | 82Z-10671 | Right | 1.402 | 0.876 | | | | 0.952 | | 0.934 | | | | 0.677 | | | 0.613 | |  |
| WT | Male | 82Z-10671 | Left | 1.454 | 0.884 | | | | 0.937 | | 0.917 | | | | 0.575 | | | 0.608 | |  |
| **Strain** | **Sex** | **No.** | **Side** | **M/1 area** | | **M/2 area** | | | | **M/3 area** | | | **M/1 elongation** | | | **M/2:M/1 area ratio** | | |  |  |
| Fgf20 | Female | 64U-G4412 | Right | 0.9992 | | 0.7556 | | | | 0.4326 | | | 1.655 | | | 0.756 | | |  |  |
| Fgf20 | Female | 64U-G4412 | Left | 1.1155 | | 0.7973 | | | | 0.3875 | | | 1.675 | | | 0.715 | | |  |  |
| Fgf20 | Female | 64U-G8045 | Right | 1.0880 | | 0.7496 | | | | 0.3409 | | | 1.560 | | | 0.689 | | |  |  |
| Fgf20 | Female | 64U-G8045 | Left | 1.1139 | | 0.7928 | | | | 0.3285 | | | 1.545 | | | 0.712 | | |  |  |
| Fgf20 | Female | 64U-G8046 | Right | 1.0662 | | 0.7848 | | | | 0.3552 | | | 1.544 | | | 0.736 | | |  |  |
| Fgf20 | Female | 64U-G8046 | Left | 1.0696 | | 0.7430 | | | | 0.3457 | | | 1.534 | | | 0.695 | | |  |  |
| Fgf20 | Female | 64U-G8049 | Right | 1.1199 | | 0.7920 | | | | 0.3891 | | | 1.546 | | | 0.707 | | |  |  |
| Fgf20 | Female | 64U-G8049 | Left | 1.1453 | | 0.8054 | | | | 0.3729 | | | 1.517 | | | 0.703 | | |  |  |
| Fgf20 | Female | 64U-G8312 | Right | 1.1277 | | 0.7456 | | | | 0.3523 | | | 1.550 | | | 0.661 | | |  |  |
| Fgf20 | Female | 64U-G8312 | Left | 1.1072 | | 0.7514 | | | | 0.3654 | | | 1.529 | | | 0.679 | | |  |  |
| Fgf20 | Male | 64U-G4411 | Right | 1.0941 | | 0.7649 | | | | 0.3782 | | | 1.581 | | | 0.699 | | |  |  |
| Fgf20 | Male | 64U-G4411 | Left | 1.1600 | | 0.7680 | | | | 0.3507 | | | 1.576 | | | 0.662 | | |  |  |
| Fgf20 | Male | 64U-G8039 | Right | 1.0579 | | 0.7947 | | | | 0.3678 | | | 1.532 | | | 0.751 | | |  |  |
| Fgf20 | Male | 64U-G8039 | Left | 1.1383 | | 0.8014 | | | | 0.3569 | | | 1.521 | | | 0.704 | | |  |  |
| Fgf20 | Male | 64U-G8040 | Right | 1.0986 | | 0.7802 | | | | 0.3369 | | | 1.468 | | | 0.710 | | |  |  |
| Fgf20 | Male | 64U-G8040 | Left | 1.1160 | | 0.8169 | | | | 0.3342 | | | 1.552 | | | 0.732 | | |  |  |
| Fgf20 | Male | 64U-G8041 | Right | 1.1409 | | 0.7855 | | | | 0.3186 | | | 1.564 | | | 0.688 | | |  |  |
| Fgf20 | Male | 64U-G8041 | Left | 1.1695 | | 0.8064 | | | | 0.3878 | | | 1.524 | | | 0.690 | | |  |  |
| Fgf20 | Male | 64U-G8042 | Right | 1.1137 | | 0.7874 | | | | 0.3479 | | | 1.560 | | | 0.707 | | |  |  |
| Fgf20 | Male | 64U-G8042 | Left | 1.0885 | | 0.7788 | | | | 0.3593 | | | 1.557 | | | 0.715 | | |  |  |
| Fgf20 | Male | 64U-G8044 | Right | 1.1274 | | 0.7812 | | | | 0.3731 | | | 1.629 | | | 0.693 | | |  |  |
| Fgf20 | Male | 64U-G8044 | Left | 1.1165 | | 0.8244 | | | | 0.3568 | | | 1.632 | | | 0.738 | | |  |  |
| WT | Female | 82Z-10501 | Right | 1.1775 | | 0.8473 | | | | 0.3786 | | | 1.705 | | | 0.720 | | |  |  |
| WT | Female | 82Z-10501 | Left | 1.2680 | | 0.8253 | | | | 0.3893 | | | 1.626 | | | 0.651 | | |  |  |
| WT | Female | 82Z-10510 | Right | 1.2220 | | 0.8873 | | | | 0.4038 | | | 1.687 | | | 0.726 | | |  |  |
| WT | Female | 82Z-10510 | Left | 1.1999 | | 0.8742 | | | | 0.3680 | | | 1.657 | | | 0.729 | | |  |  |
| WT | Female | 82Z-10511 | Right | 1.1454 | | 0.8628 | | | | 0.3574 | | | 1.510 | | | 0.753 | | |  |  |
| WT | Female | 82Z-10511 | Left | 1.2043 | | 0.8289 | | | | 0.3736 | | | 1.632 | | | 0.688 | | |  |  |
| WT | Female | 82Z-10530 | Right | 1.1961 | | 0.8602 | | | | 0.3788 | | | 1.562 | | | 0.719 | | |  |  |
| WT | Female | 82Z-10530 | Left | 1.2292 | | 0.8920 | | | | 0.3770 | | | 1.643 | | | 0.726 | | |  |  |
| WT | Female | 82Z-10531 | Right | 1.2169 | | 0.8892 | | | | 0.3941 | | | 1.615 | | | 0.731 | | |  |  |
| WT | Female | 82Z-10531 | Left | 1.2128 | | 0.8587 | | | | 0.3787 | | | 1.703 | | | 0.708 | | |  |  |
| WT | Female | 82Z-10532 | Right | 1.2283 | | 0.9063 | | | | 0.3795 | | | 1.642 | | | 0.738 | | |  |  |
| WT | Female | 82Z-10532 | Left | 1.2019 | | 0.8912 | | | | 0.4025 | | | 1.566 | | | 0.741 | | |  |  |
| WT | Male | 82Z-10667 | Right | 1.2479 | | 0.9311 | | | | 0.3909 | | | 1.652 | | | 0.746 | | |  |  |
| WT | Male | 82Z-10667 | Left | 1.2464 | | 0.8949 | | | | 0.4012 | | | 1.577 | | | 0.718 | | |  |  |
| WT | Male | 82Z-10668 | Right | 1.2420 | | 0.8930 | | | | 0.4142 | | | 1.637 | | | 0.719 | | |  |  |
| WT | Male | 82Z-10668 | Left | 1.2750 | | 0.8996 | | | | 0.4091 | | | 1.588 | | | 0.706 | | |  |  |
| WT | Male | 82Z-10669 | Right | 1.2542 | | 0.9070 | | | | 0.3903 | | | 1.688 | | | 0.723 | | |  |  |
| WT | Male | 82Z-10669 | Left | 1.2882 | | 0.8920 | | | | 0.4227 | | | 1.630 | | | 0.692 | | |  |  |
| WT | Male | 82Z-10670 | Right | 1.2848 | | 0.8949 | | | | 0.4088 | | | 1.618 | | | 0.697 | | |  |  |
| WT | Male | 82Z-10670 | Left | 1.2610 | | 0.8901 | | | | 0.4125 | | | 1.599 | | | 0.706 | | |  |  |
| WT | Male | 82Z-10671 | Right | 1.2282 | | 0.8892 | | | | 0.4150 | | | 1.600 | | | 0.724 | | |  |  |
| WT | Male | 82Z-10671 | Left | 1.2853 | | 0.8592 | | | | 0.3496 | | | 1.645 | | | 0.668 | | |  |  |
| **Strain** | **Sex** | **No.** | **Side** | **Enamel mineralization** | | | **Enamel thickness** | | | | | **Crown dentine mineralization** | | | | | **Crown dentine thickness** | | | |
| Fgf20 | Female | 64U-G4412 | Right | 0.1031 | | | 0.1058 | | | | | 0.0779 | | | | | 0.1569 | | | |
| Fgf20 | Female | 64U-G4412 | Left | 0.1055 | | | 0.1041 | | | | | 0.0807 | | | | | 0.1629 | | | |
| Fgf20 | Female | 64U-G8045 | Right | 0.1044 | | | 0.1025 | | | | | 0.0796 | | | | | 0.1583 | | | |
| Fgf20 | Female | 64U-G8045 | Left | 0.1045 | | | 0.1037 | | | | | 0.0802 | | | | | 0.1593 | | | |
| Fgf20 | Female | 64U-G8046 | Right | 0.1042 | | | 0.1029 | | | | | 0.0790 | | | | | 0.1585 | | | |
| Fgf20 | Female | 64U-G8046 | Left | 0.1046 | | | 0.1040 | | | | | 0.0794 | | | | | 0.1573 | | | |
| Fgf20 | Female | 64U-G8049 | Right | 0.1054 | | | 0.1042 | | | | | 0.0798 | | | | | 0.1687 | | | |
| Fgf20 | Female | 64U-G8049 | Left | 0.1057 | | | 0.1058 | | | | | 0.0807 | | | | | 0.1594 | | | |
| Fgf20 | Female | 64U-G8312 | Right | 0.1040 | | | 0.1074 | | | | | 0.0784 | | | | | 0.1561 | | | |
| Fgf20 | Female | 64U-G8312 | Left | 0.1039 | | | 0.1074 | | | | | 0.0786 | | | | | 0.1513 | | | |
| Fgf20 | Male | 64U-G4411 | Right | 0.1039 | | | 0.1046 | | | | | 0.0785 | | | | | 0.1651 | | | |
| Fgf20 | Male | 64U-G4411 | Left | 0.1036 | | | 0.1047 | | | | | 0.0791 | | | | | 0.1616 | | | |
| Fgf20 | Male | 64U-G8039 | Right | 0.1038 | | | 0.1030 | | | | | 0.0795 | | | | | 0.1599 | | | |
| Fgf20 | Male | 64U-G8039 | Left | 0.1043 | | | 0.1057 | | | | | 0.0804 | | | | | 0.1604 | | | |
| Fgf20 | Male | 64U-G8040 | Right | 0.1048 | | | 0.1044 | | | | | 0.0796 | | | | | 0.1626 | | | |
| Fgf20 | Male | 64U-G8040 | Left | 0.1051 | | | 0.1033 | | | | | 0.0807 | | | | | 0.1648 | | | |
| Fgf20 | Male | 64U-G8041 | Right | 0.1038 | | | 0.1027 | | | | | 0.0787 | | | | | 0.1613 | | | |
| Fgf20 | Male | 64U-G8041 | Left | 0.1053 | | | 0.1051 | | | | | 0.0803 | | | | | 0.1636 | | | |
| Fgf20 | Male | 64U-G8042 | Right | 0.1047 | | | 0.1018 | | | | | 0.0794 | | | | | 0.1561 | | | |
| Fgf20 | Male | 64U-G8042 | Left | 0.1043 | | | 0.1026 | | | | | 0.0804 | | | | | 0.1597 | | | |
| Fgf20 | Male | 64U-G8044 | Right | 0.1041 | | | 0.1042 | | | | | 0.0786 | | | | | 0.1568 | | | |
| Fgf20 | Male | 64U-G8044 | Left | 0.1044 | | | 0.1049 | | | | | 0.0796 | | | | | 0.1574 | | | |
| WT | Female | 82Z-10501 | Right | 0.1038 | | | 0.1039 | | | | | 0.0792 | | | | | 0.1677 | | | |
| WT | Female | 82Z-10501 | Left | 0.1031 | | | 0.1056 | | | | | 0.0792 | | | | | 0.1661 | | | |
| WT | Female | 82Z-10510 | Right | 0.1067 | | | 0.1073 | | | | | 0.0813 | | | | | 0.1744 | | | |
| WT | Female | 82Z-10510 | Left | 0.1064 | | | 0.1082 | | | | | 0.0811 | | | | | 0.1711 | | | |
| WT | Female | 82Z-10511 | Right | 0.1031 | | | 0.1045 | | | | | 0.0787 | | | | | 0.1641 | | | |
| WT | Female | 82Z-10511 | Left | 0.1036 | | | 0.1084 | | | | | 0.0796 | | | | | 0.1641 | | | |
| WT | Female | 82Z-10530 | Right | 0.1049 | | | 0.1055 | | | | | 0.0803 | | | | | 0.1666 | | | |
| WT | Female | 82Z-10530 | Left | 0.1054 | | | 0.1066 | | | | | 0.0813 | | | | | 0.1724 | | | |
| WT | Female | 82Z-10531 | Right | 0.1048 | | | 0.1081 | | | | | 0.0800 | | | | | 0.1708 | | | |
| WT | Female | 82Z-10531 | Left | 0.1044 | | | 0.1040 | | | | | 0.0806 | | | | | 0.1736 | | | |
| WT | Female | 82Z-10532 | Right | 0.1039 | | | 0.1060 | | | | | 0.0787 | | | | | 0.1743 | | | |
| WT | Female | 82Z-10532 | Left | 0.1028 | | | 0.1063 | | | | | 0.0790 | | | | | 0.1688 | | | |
| WT | Male | 82Z-10667 | Right | 0.1056 | | | 0.1070 | | | | | 0.0808 | | | | | 0.1729 | | | |
| WT | Male | 82Z-10667 | Left | 0.1044 | | | 0.1048 | | | | | 0.0809 | | | | | 0.1745 | | | |
| WT | Male | 82Z-10668 | Right | 0.1036 | | | 0.1055 | | | | | 0.0800 | | | | | 0.1761 | | | |
| WT | Male | 82Z-10668 | Left | 0.1037 | | | 0.1079 | | | | | 0.0804 | | | | | 0.1741 | | | |
| WT | Male | 82Z-10669 | Right | 0.1062 | | | 0.1036 | | | | | 0.0812 | | | | | 0.1763 | | | |
| WT | Male | 82Z-10669 | Left | 0.1053 | | | 0.1061 | | | | | 0.0819 | | | | | 0.1725 | | | |
| WT | Male | 82Z-10670 | Right | 0.1047 | | | 0.1091 | | | | | 0.0800 | | | | | 0.1754 | | | |
| WT | Male | 82Z-10670 | Left | 0.1049 | | | 0.1057 | | | | | 0.0807 | | | | | 0.1774 | | | |
| WT | Male | 82Z-10671 | Right | 0.1046 | | | 0.1032 | | | | | 0.0808 | | | | | 0.1710 | | | |
| WT | Male | 82Z-10671 | Left | 0.1052 | | | 0.1066 | | | | | 0.0815 | | | | | 0.1720 | | | |
| Strain | Sex | No. | Side | Crown pulp spatial diversity | | | Relative crown pulp volume | | | | | Root dentine mineralization | | | | | Root dentine thickness | | | |
| Fgf20 | Female | 64U-G4412 | Right | 23.6366 | | | 0.2016 | | | | | 0.0704 | | | | | 0.1616 | | | |
| Fgf20 | Female | 64U-G4412 | Left | 24.1459 | | | 0.1860 | | | | | 0.0720 | | | | | 0.1705 | | | |
| Fgf20 | Female | 64U-G8045 | Right | 24.2580 | | | 0.1917 | | | | | 0.0715 | | | | | 0.1577 | | | |
| Fgf20 | Female | 64U-G8045 | Left | 24.1056 | | | 0.1928 | | | | | 0.0719 | | | | | 0.1655 | | | |
| Fgf20 | Female | 64U-G8046 | Right | 24.3205 | | | 0.1948 | | | | | 0.0714 | | | | | 0.1571 | | | |
| Fgf20 | Female | 64U-G8046 | Left | 23.5776 | | | 0.1992 | | | | | 0.0713 | | | | | 0.1600 | | | |
| Fgf20 | Female | 64U-G8049 | Right | 25.7193 | | | 0.1652 | | | | | 0.0713 | | | | | 0.1740 | | | |
| Fgf20 | Female | 64U-G8049 | Left | 25.7902 | | | 0.1768 | | | | | 0.0721 | | | | | 0.1781 | | | |
| Fgf20 | Female | 64U-G8312 | Right | 23.4019 | | | 0.1991 | | | | | 0.0707 | | | | | 0.1647 | | | |
| Fgf20 | Female | 64U-G8312 | Left | 23.1993 | | | 0.2051 | | | | | 0.0710 | | | | | 0.1671 | | | |
| Fgf20 | Male | 64U-G4411 | Right | 24.5474 | | | 0.1839 | | | | | 0.0701 | | | | | 0.1685 | | | |
| Fgf20 | Male | 64U-G4411 | Left | 23.8859 | | | 0.1917 | | | | | 0.0711 | | | | | 0.1658 | | | |
| Fgf20 | Male | 64U-G8039 | Right | 24.1217 | | | 0.1919 | | | | | 0.0710 | | | | | 0.1655 | | | |
| Fgf20 | Male | 64U-G8039 | Left | 24.6757 | | | 0.1830 | | | | | 0.0721 | | | | | 0.1703 | | | |
| Fgf20 | Male | 64U-G8040 | Right | 24.9828 | | | 0.1796 | | | | | 0.0717 | | | | | 0.1724 | | | |
| Fgf20 | Male | 64U-G8040 | Left | 24.5793 | | | 0.1772 | | | | | 0.0728 | | | | | 0.1769 | | | |
| Fgf20 | Male | 64U-G8041 | Right | 23.8314 | | | 0.1883 | | | | | 0.0716 | | | | | 0.1721 | | | |
| Fgf20 | Male | 64U-G8041 | Left | 23.6170 | | | 0.1836 | | | | | 0.0727 | | | | | 0.1763 | | | |
| Fgf20 | Male | 64U-G8042 | Right | 23.8595 | | | 0.2019 | | | | | 0.0707 | | | | | 0.1629 | | | |
| Fgf20 | Male | 64U-G8042 | Left | 24.2725 | | | 0.1853 | | | | | 0.0726 | | | | | 0.1741 | | | |
| Fgf20 | Male | 64U-G8044 | Right | 23.4186 | | | 0.2035 | | | | | 0.0703 | | | | | 0.1646 | | | |
| Fgf20 | Male | 64U-G8044 | Left | 24.2055 | | | 0.1968 | | | | | 0.0719 | | | | | 0.1708 | | | |
| WT | Female | 82Z-10501 | Right | 26.3716 | | | 0.1705 | | | | | 0.0711 | | | | | 0.1747 | | | |
| WT | Female | 82Z-10501 | Left | 25.0781 | | | 0.1785 | | | | | 0.0711 | | | | | 0.1762 | | | |
| WT | Female | 82Z-10510 | Right | 25.8174 | | | 0.1662 | | | | | 0.0726 | | | | | 0.1804 | | | |
| WT | Female | 82Z-10510 | Left | 24.9775 | | | 0.1715 | | | | | 0.0728 | | | | | 0.1767 | | | |
| WT | Female | 82Z-10511 | Right | 25.0102 | | | 0.1772 | | | | | 0.0701 | | | | | 0.1656 | | | |
| WT | Female | 82Z-10511 | Left | 24.1260 | | | 0.1850 | | | | | 0.0710 | | | | | 0.1720 | | | |
| WT | Female | 82Z-10530 | Right | 25.4569 | | | 0.1733 | | | | | 0.0714 | | | | | 0.1719 | | | |
| WT | Female | 82Z-10530 | Left | 26.3759 | | | 0.1635 | | | | | 0.0723 | | | | | 0.1714 | | | |
| WT | Female | 82Z-10531 | Right | 24.6170 | | | 0.1769 | | | | | 0.0711 | | | | | 0.1753 | | | |
| WT | Female | 82Z-10531 | Left | 25.1690 | | | 0.1672 | | | | | 0.0718 | | | | | 0.1796 | | | |
| WT | Female | 82Z-10532 | Right | 25.1279 | | | 0.1711 | | | | | 0.0701 | | | | | 0.1684 | | | |
| WT | Female | 82Z-10532 | Left | 24.5954 | | | 0.1767 | | | | | 0.0707 | | | | | 0.1735 | | | |
| WT | Male | 82Z-10667 | Right | 25.1007 | | | 0.1741 | | | | | 0.0716 | | | | | 0.1770 | | | |
| WT | Male | 82Z-10667 | Left | 24.3361 | | | 0.1718 | | | | | 0.0724 | | | | | 0.1934 | | | |
| WT | Male | 82Z-10668 | Right | 24.3183 | | | 0.1691 | | | | | 0.0711 | | | | | 0.1825 | | | |
| WT | Male | 82Z-10668 | Left | 25.0853 | | | 0.1701 | | | | | 0.0716 | | | | | 0.1893 | | | |
| WT | Male | 82Z-10669 | Right | 24.3300 | | | 0.1677 | | | | | 0.0727 | | | | | 0.1834 | | | |
| WT | Male | 82Z-10669 | Left | 26.5542 | | | 0.1615 | | | | | 0.0732 | | | | | 0.1903 | | | |
| WT | Male | 82Z-10670 | Right | 24.6765 | | | 0.1728 | | | | | 0.0719 | | | | | 0.1862 | | | |
| WT | Male | 82Z-10670 | Left | 25.3799 | | | 0.1632 | | | | | 0.0735 | | | | | 0.1879 | | | |
| WT | Male | 82Z-10671 | Right | 26.1948 | | | 0.1659 | | | | | 0.0723 | | | | | 0.1883 | | | |
| WT | Male | 82Z-10671 | Left | 26.3911 | | | 0.1642 | | | | | 0.0727 | | | | | 0.1915 | | | |
| **Strain** | **Sex** | **No.** | **Side** | **Root pulp spatial diversity** | | | | **Relative root pulp volume** | | | | | |  |  |  |  |  |  |  |
| Fgf20 | Female | 64U-G4412 | Right | 32.4450 | | | | 0.1197 | | | | | |  |  |  |  |  |  |  |
| Fgf20 | Female | 64U-G4412 | Left | 34.4799 | | | | 0.1076 | | | | | |  |  |  |  |  |  |  |
| Fgf20 | Female | 64U-G8045 | Right | 36.2887 | | | | 0.1084 | | | | | |  |  |  |  |  |  |  |
| Fgf20 | Female | 64U-G8045 | Left | 36.5796 | | | | 0.0992 | | | | | |  |  |  |  |  |  |  |
| Fgf20 | Female | 64U-G8046 | Right | 32.9646 | | | | 0.1221 | | | | | |  |  |  |  |  |  |  |
| Fgf20 | Female | 64U-G8046 | Left | 35.7039 | | | | 0.1156 | | | | | |  |  |  |  |  |  |  |
| Fgf20 | Female | 64U-G8049 | Right | 32.7490 | | | | 0.1044 | | | | | |  |  |  |  |  |  |  |
| Fgf20 | Female | 64U-G8049 | Left | 35.0195 | | | | 0.0991 | | | | | |  |  |  |  |  |  |  |
| Fgf20 | Female | 64U-G8312 | Right | 32.5312 | | | | 0.1177 | | | | | |  |  |  |  |  |  |  |
| Fgf20 | Female | 64U-G8312 | Left | 33.0010 | | | | 0.1165 | | | | | |  |  |  |  |  |  |  |
| Fgf20 | Male | 64U-G4411 | Right | 34.6266 | | | | 0.1060 | | | | | |  |  |  |  |  |  |  |
| Fgf20 | Male | 64U-G4411 | Left | 35.0793 | | | | 0.1107 | | | | | |  |  |  |  |  |  |  |
| Fgf20 | Male | 64U-G8039 | Right | 34.2545 | | | | 0.1132 | | | | | |  |  |  |  |  |  |  |
| Fgf20 | Male | 64U-G8039 | Left | 33.1726 | | | | 0.1106 | | | | | |  |  |  |  |  |  |  |
| Fgf20 | Male | 64U-G8040 | Right | 34.7954 | | | | 0.1082 | | | | | |  |  |  |  |  |  |  |
| Fgf20 | Male | 64U-G8040 | Left | 34.6121 | | | | 0.1088 | | | | | |  |  |  |  |  |  |  |
| Fgf20 | Male | 64U-G8041 | Right | 35.9341 | | | | 0.1061 | | | | | |  |  |  |  |  |  |  |
| Fgf20 | Male | 64U-G8041 | Left | 36.6385 | | | | 0.0936 | | | | | |  |  |  |  |  |  |  |
| Fgf20 | Male | 64U-G8042 | Right | 32.7868 | | | | 0.1153 | | | | | |  |  |  |  |  |  |  |
| Fgf20 | Male | 64U-G8042 | Left | 37.2436 | | | | 0.0998 | | | | | |  |  |  |  |  |  |  |
| Fgf20 | Male | 64U-G8044 | Right | 33.4257 | | | | 0.1154 | | | | | |  |  |  |  |  |  |  |
| Fgf20 | Male | 64U-G8044 | Left | 33.2903 | | | | 0.1110 | | | | | |  |  |  |  |  |  |  |
| WT | Female | 82Z-10501 | Right | 34.3718 | | | | 0.1036 | | | | | |  |  |  |  |  |  |  |
| WT | Female | 82Z-10501 | Left | 38.4470 | | | | 0.0924 | | | | | |  |  |  |  |  |  |  |
| WT | Female | 82Z-10510 | Right | 38.8019 | | | | 0.0894 | | | | | |  |  |  |  |  |  |  |
| WT | Female | 82Z-10510 | Left | 37.8937 | | | | 0.0905 | | | | | |  |  |  |  |  |  |  |
| WT | Female | 82Z-10511 | Right | 37.0781 | | | | 0.1092 | | | | | |  |  |  |  |  |  |  |
| WT | Female | 82Z-10511 | Left | 37.8690 | | | | 0.0937 | | | | | |  |  |  |  |  |  |  |
| WT | Female | 82Z-10530 | Right | 36.3471 | | | | 0.1015 | | | | | |  |  |  |  |  |  |  |
| WT | Female | 82Z-10530 | Left | 36.6498 | | | | 0.1012 | | | | | |  |  |  |  |  |  |  |
| WT | Female | 82Z-10531 | Right | 36.7354 | | | | 0.0995 | | | | | |  |  |  |  |  |  |  |
| WT | Female | 82Z-10531 | Left | 38.8674 | | | | 0.0864 | | | | | |  |  |  |  |  |  |  |
| WT | Female | 82Z-10532 | Right | 34.7723 | | | | 0.1040 | | | | | |  |  |  |  |  |  |  |
| WT | Female | 82Z-10532 | Left | 36.0789 | | | | 0.0977 | | | | | |  |  |  |  |  |  |  |
| WT | Male | 82Z-10667 | Right | 36.6005 | | | | 0.0968 | | | | | |  |  |  |  |  |  |  |
| WT | Male | 82Z-10667 | Left | 40.2744 | | | | 0.0767 | | | | | |  |  |  |  |  |  |  |
| WT | Male | 82Z-10668 | Right | 36.9868 | | | | 0.0951 | | | | | |  |  |  |  |  |  |  |
| WT | Male | 82Z-10668 | Left | 38.6025 | | | | 0.0850 | | | | | |  |  |  |  |  |  |  |
| WT | Male | 82Z-10669 | Right | 39.3176 | | | | 0.0864 | | | | | |  |  |  |  |  |  |  |
| WT | Male | 82Z-10669 | Left | 38.7793 | | | | 0.0850 | | | | | |  |  |  |  |  |  |  |
| WT | Male | 82Z-10670 | Right | 37.7857 | | | | 0.0908 | | | | | |  |  |  |  |  |  |  |
| WT | Male | 82Z-10670 | Left | 36.1303 | | | | 0.0972 | | | | | |  |  |  |  |  |  |  |
| WT | Male | 82Z-10671 | Right | 37.9027 | | | | 0.0866 | | | | | |  |  |  |  |  |  |  |
| WT | Male | 82Z-10671 | Left | 38.7161 | | | | 0.0830 | | | | | |  |  |  |  |  |  |  |

**Table S4:** Means and Standard deviations for all variables in Table S3.

| **Variable** | **WT Females** (N = 12) | | | | | **Fgf20 Females** (N = 10) | | | |
| --- | --- | --- | --- | --- | --- | --- | --- | --- | --- |
|  | ***Mean*** | | | ***Std.Dev.*** | | ***Mean*** | | ***Std.Dev.*** | |
| *Length of M/1* | 1.4029 | | | 0.0359 | | 1.3089 | | 0.0253 | |
| *Width of M/1* | 0.8616 | | | 0.0150 | | 0.8367 | | 0.0256 | |
| *Length of M/2* | 0.9407 | | | 0.0256 | | 0.8672 | | 0.0279 | |
| *Width of M/2* | 0.9235 | | | 0.0184 | | 0.8901 | | 0.0174 | |
| *Length of M/3* | 0.5936 | | | 0.0285 | | 0.5896 | | 0.0383 | |
| *Width of M/3* | 0.6436 | | | 0.0132 | | 0.6222 | | 0.0225 | |
| *M/1 area* | 1.2085 | | | 0.0299 | | 1.0952 | | 0.0420 | |
| *M/2 area* | 0.8686 | | | 0.0259 | | 0.7718 | | 0.0247 | |
| *M/3 area* | 0.3818 | | | 0.0136 | | 0.3670 | | 0.0302 | |
| *M/1 elongation* | 1.6290 | | | 0.0592 | | 1.5656 | | 0.0540 | |
| *M/2:M/1 area ratio* | 0.7191 | | | 0.0271 | | 0.7053 | | 0.0273 | |
| *Enamel mineralization* | 0.1044 | | | 0.0013 | | 0.1045 | | 0.0008 | |
| *Enamel thickness* | 0.1062 | | | 0.0016 | | 0.1048 | | 0.0017 | |
| *Crown dentine mineralization* | 0.0799 | | | 0.0010 | | 0.0794 | | 0.0010 | |
| *Crown dentine thickness* | 0.1695 | | | 0.0038 | | 0.1589 | | 0.0045 | |
| *Crown pulp spatial diversity* | 25.2269 | | | 0.6849 | | 24.2155 | | 0.8949 | |
| *Relative crown pulp volume* | 0.1731 | | | 0.0061 | | 0.1912 | | 0.0122 | |
| *Root dentine mineralization* | 0.0713 | | | 0.0009 | | 0.0714 | | 0.0006 | |
| *Root dentine thickness* | 0.1738 | | | 0.0043 | | 0.1656 | | 0.0070 | |
| *Root pulp spatial diversity* | 36.9927 | | | 1.4686 | | 34.1762 | | 1.6318 | |
| *Relative root pulp volume* | 0.0974 | | | 0.0069 | | 0.1110 | | 0.0084 | |
| **Variable** |  | **WT Males** (N = 10) | | | **Fgf20 Males** (N = 12) | | | |  |
|  |  | ***Mean*** | ***Std.Dev.*** | | ***Mean*** | | ***Std.Dev.*** | |  |
| *Length of M/1* |  | 1.4309 | 0.0196 | | 1.3198 | | 0.0282 | |  |
| *Width of M/1* |  | 0.8815 | 0.0112 | | 0.8474 | | 0.0162 | |  |
| *Length of M/2* |  | 0.9482 | 0.0144 | | 0.8933 | | 0.0238 | |  |
| *Width of M/2* |  | 0.9440 | 0.0130 | | 0.8855 | | 0.0146 | |  |
| *Length of M/3* |  | 0.6181 | 0.0256 | | 0.5779 | | 0.0293 | |  |
| *Width of M/3* |  | 0.6496 | 0.0260 | | 0.6157 | | 0.0216 | |  |
| *M/1 area* |  | 1.2613 | 0.0210 | | 1.1184 | | 0.0315 | |  |
| *M/2 area* |  | 0.8951 | 0.0177 | | 0.7908 | | 0.0185 | |  |
| *M/3 area* |  | 0.4014 | 0.0210 | | 0.3557 | | 0.0197 | |  |
| *M/1 elongation* |  | 1.6236 | 0.0337 | | 1.5580 | | 0.0454 | |  |
| *M/2:M/1 area ratio* |  | 0.7099 | 0.0213 | | 0.7075 | | 0.0245 | |  |
| *Enamel mineralization* |  | 0.1048 | 0.0008 | | 0.1043 | | 0.0005 | |  |
| *Enamel thickness* |  | 0.1059 | 0.0018 | | 0.1039 | | 0.0012 | |  |
| *Crown dentine mineralization* |  | 0.0808 | 0.0006 | | 0.0796 | | 0.0008 | |  |
| *Crown dentine thickness* |  | 0.1742 | 0.0021 | | 0.1608 | | 0.0030 | |  |
| *Crown pulp spatial diversity* |  | 25.2367 | 0.8717 | | 24.1664 | | 0.4673 | |  |
| *Relative crown pulp volume* |  | 0.1680 | 0.0043 | | 0.1889 | | 0.0084 | |  |
| *Root dentine mineralization* |  | 0.0723 | 0.0008 | | 0.0716 | | 0.0009 | |  |
| *Root dentine thickness* |  | 0.1870 | 0.0049 | | 0.1700 | | 0.0046 | |  |
| *Root pulp spatial diversity* |  | 38.1096 | 1.2827 | | 34.6550 | | 1.4044 | |  |
| *Relative root pulp volume* |  | 0.0882 | 0.0066 | | 0.1082 | | 0.0063 | |  |

**Table S5:** Results of the Kruskal-Wallis non-parametric test with a post-hoc multiple comparison test was used to detect the presence of significant differences among the four groups, i.e. WT females, KO females, WT males, and KO males. Relevant results comparing WT and KO mice highlighted in yellow. Significant results (p < 0.05) in red.

| Kruskal-Wallis ANOVA by Ranks; **M/1 area** Independent (grouping) variable: Group **Kruskal-Wallis test: H ( 3, N= 44) =35,24323 p < 0.0001** |  |  |  |  |
| --- | --- | --- | --- | --- |
|  | **Fgf20.F** | **Fgf20.M** | **WT.F** | **WT.M** |
| **Fgf20.F** |  | 1.000000 | 0.002698 | 0.000003 |
| **Fgf20.M** | 1.000000 |  | 0.015202 | 0.000021 |
| **WT.F** | 0.002698 | 0.015202 |  | 0.466759 |
| **WT.M** | 0.000003 | 0.000021 | 0.466759 |  |
|  |  |  |  |  |
| Kruskal-Wallis ANOVA by Ranks; **M/2 area** Independent (grouping) variable: Group **Kruskal-Wallis test: H ( 3, N= 44) =34,73406 p < 0.0001** |  |  |  |  |
|  | **Fgf20.F** | **Fgf20.M** | **WT.F** | **WT.M** |
| **Fgf20.F** |  | 1.000000 | 0.000792 | 0.000004 |
| **Fgf20.M** | 1.000000 |  | 0.009138 | 0.000074 |
| **WT.F** | 0.000792 | 0.009138 |  | 1.000000 |
| **WT.M** | 0.000004 | 0.000074 | 1.000000 |  |
|  |  |  |  |  |
| Kruskal-Wallis ANOVA by Ranks; **M/3 area** Independent (grouping) variable: Group **Kruskal-Wallis test: H ( 3, N= 44) =19,32545 p = 0.0002** |  |  |  |  |
|  | **Fgf20.F** | **Fgf20.M** | **WT.F** | **WT.M** |
| **Fgf20.F** |  | 1.000000 | 0.577111 | 0.010368 |
| **Fgf20.M** | 1.000000 |  | 0.060257 | 0.000290 |
| **WT.F** | 0.577111 | 0.060257 |  | 0.645579 |
| **WT.M** | 0.010368 | 0.000290 | 0.645579 |  |
|  |  |  |  |  |
| Kruskal-Wallis ANOVA by Ranks; **M/1 elongation** Independent (grouping) variable: Group **Kruskal-Wallis test: H ( 3, N= 44) =14,46293 p = 0.0023** |  |  |  |  |
|  | **Fgf20.F** | **Fgf20.M** | **WT.F** | **WT.M** |
| **Fgf20.F** |  | 1.000000 | 0.047223 | 0.069592 |
| **Fgf20.M** | 1.000000 |  | 0.026689 | 0.042755 |
| **WT.F** | 0.047223 | 0.026689 |  | 1.000000 |
| **WT.M** | 0.069592 | 0.042755 | 1.000000 |  |
|  |  |  |  |  |
| Kruskal-Wallis ANOVA by Ranks; **M/2:M/1 area ratio** Independent (grouping) variable: Group **Kruskal-Wallis test: H ( 3, N= 44) =3,624141 p = 0.3050** |  |  |  |  |
|  |  |  |  |  |
| Kruskal-Wallis ANOVA by Ranks; **Enamel mineralization** Independent (grouping) variable: Group **Kruskal-Wallis test: H ( 3, N= 44) =2,397071 p = 0.4942** |  |  |  |  |
|  |  |  |  |  |
| Kruskal-Wallis ANOVA by Ranks; **Enamel thickness** Independent (grouping) variable: Group **Kruskal-Wallis test: H ( 3, N= 44) =11,25364 p = 0.0104** |  |  |  |  |
|  | **Fgf20.F** | **Fgf20.M** | **WT.F** | **WT.M** |
| **Fgf20.F** |  | 1.000000 | 0.414218 | 0.951187 |
| **Fgf20.M** | 1.000000 |  | 0.016018 | 0.070777 |
| **WT.F** | 0.414218 | 0.016018 |  | 1.000000 |
| **WT.M** | 0.951187 | 0.070777 | 1.000000 |  |
|  |  |  |  |  |
| Kruskal-Wallis ANOVA by Ranks; **Crown dentine mineralization** Independent (grouping) variable: Group **Kruskal-Wallis test: H ( 3, N= 44) =13,09444 p = 0.0044** |  |  |  |  |
|  | **Fgf20.F** | **Fgf20.M** | **WT.F** | **WT.M** |
| **Fgf20.F** |  | 1.000000 | 1.000000 | 0.008667 |
| **Fgf20.M** | 1.000000 |  | 1.000000 | 0.010586 |
| **WT.F** | 1.000000 | 1.000000 |  | 0.153162 |
| **WT.M** | 0.008667 | 0.010586 | 0.153162 |  |
|  |  |  |  |  |
| Kruskal-Wallis ANOVA by Ranks; **Crown dentine thickness** Independent (grouping) variable: Group **Kruskal-Wallis test: H ( 3, N= 44) =32,91747 p < 0.0001** |  |  |  |  |
|  | **Fgf20.F** | **Fgf20.M** | **WT.F** | **WT.M** |
| **Fgf20.F** |  | 1.000000 | 0.002406 | 0.000006 |
| **Fgf20.M** | 1.000000 |  | 0.019703 | 0.000074 |
| **WT.F** | 0.002406 | 0.019703 |  | 0.698914 |
| **WT.M** | 0.000006 | 0.000074 | 0.698914 |  |
|  |  |  |  |  |
| Kruskal-Wallis ANOVA by Ranks; **Crown pulp spatial diversity** Independent (grouping) variable: Group **Kruskal-Wallis test: H ( 3, N= 44) =18,25404 p = 0.0004** |  |  |  |  |
|  | **Fgf20.F** | **Fgf20.M** | **WT.F** | **WT.M** |
| **Fgf20.F** |  | 1.000000 | 0.016855 | 0.027284 |
| **Fgf20.M** | 1.000000 |  | 0.008416 | 0.015106 |
| **WT.F** | 0.016855 | 0.008416 |  | 1.000000 |
| **WT.M** | 0.027284 | 0.015106 | 1.000000 |  |
|  |  |  |  |  |
| Kruskal-Wallis ANOVA by Ranks; **Relative crown pulp volume** Independent (grouping) variable: Group **Kruskal-Wallis test: H ( 3, N= 44) =26,66566 p < 0.0001** |  |  |  |  |
|  | **Fgf20.F** | **Fgf20.M** | **WT.F** | **WT.M** |
| **Fgf20.F** |  | 1.000000 | 0.013120 | 0.000278 |
| **Fgf20.M** | 1.000000 |  | 0.012301 | 0.000217 |
| **WT.F** | 0.013120 | 0.012301 |  | 1.000000 |
| **WT.M** | 0.000278 | 0.000217 | 1.000000 |  |
|  |  |  |  |  |
| Kruskal-Wallis ANOVA by Ranks; **Root dentine mineralization** Independent (grouping) variable: Group **Kruskal-Wallis test: H ( 3, N= 44) =7,967980 p = 0.0467** |  |  |  |  |
|  | **Fgf20.F** | **Fgf20.M** | **WT.F** | **WT.M** |
| **Fgf20.F** |  | 1.000000 | 1.000000 | 0.112626 |
| **Fgf20.M** | 1.000000 |  | 1.000000 | 0.369155 |
| **WT.F** | 1.000000 | 1.000000 |  | 0.064353 |
| **WT.M** | 0.112626 | 0.369155 | 0.064353 |  |
|  |  |  |  |  |
| Kruskal-Wallis ANOVA by Ranks; **Root dentine thickness** Independent (grouping) variable: Group **Kruskal-Wallis test: H ( 3, N= 44) =27,06414 p < 0.0001** |  |  |  |  |
|  | **Fgf20.F** | **Fgf20.M** | **WT.F** | **WT.M** |
| **Fgf20.F** |  | 1.000000 | 0.139356 | 0.000006 |
| **Fgf20.M** | 1.000000 |  | 1.000000 | 0.000326 |
| **WT.F** | 0.139356 | 1.000000 |  | 0.027120 |
| **WT.M** | 0.000006 | 0.000326 | 0.027120 |  |
|  |  |  |  |  |
| Kruskal-Wallis ANOVA by Ranks; **Root pulp spatial diversity** Independent (grouping) variable: Group **Kruskal-Wallis test: H ( 3, N= 44) =24,57172 p < 0.0001** |  |  |  |  |
|  | **Fgf20.F** | **Fgf20.M** | **WT.F** | **WT.M** |
| **Fgf20.F** |  | 1.000000 | 0.007740 | 0.000206 |
| **Fgf20.M** | 1.000000 |  | 0.041419 | 0.001373 |
| **WT.F** | 0.007740 | 0.041419 |  | 1.000000 |
| **WT.M** | 0.000206 | 0.001373 | 1.000000 |  |
|  |  |  |  |  |
| Kruskal-Wallis ANOVA by Ranks; **Relative root pulp volume** Independent (grouping) variable: Group **Kruskal-Wallis test: H ( 3, N= 44) =27,05232 p < 0.0001** |  |  |  |  |
|  | **Fgf20.F** | **Fgf20.M** | **WT.F** | **WT.M** |
| **Fgf20.F** |  | 1.000000 | 0.025613 | 0.000050 |
| **Fgf20.M** | 1.000000 |  | 0.072278 | 0.000167 |
| **WT.F** | 0.025613 | 0.072278 |  | 0.434043 |
| **WT.M** | 0.000050 | 0.000167 | 0.434043 |  |

**
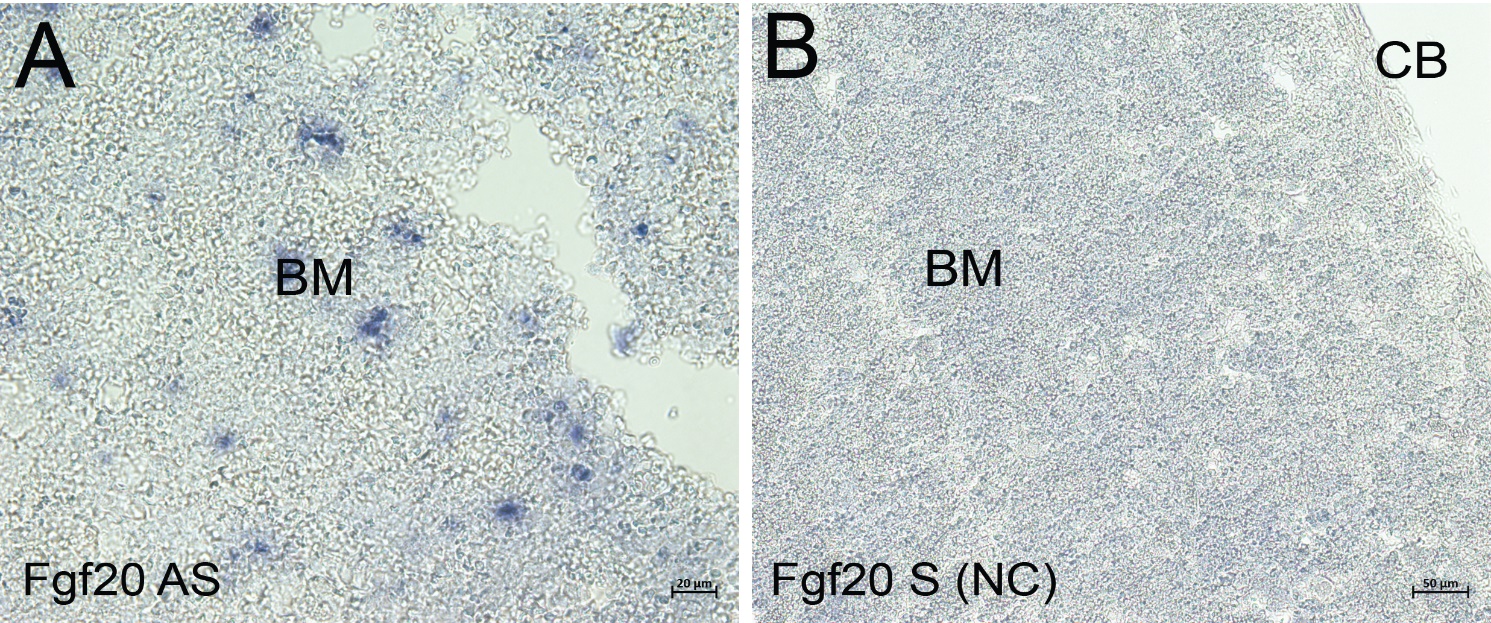
**

**Figure S1: RNA *in situ* hybridization in long bone.** Sections of the femoral diaphyses were processed for ISH with DIG-labeled Fgf20 probe. Highly positive cells (A) and negative control staining with Fgf20 sense probe (B). BM, bone marrow; CB, cortical bone. Scale bar shown (20 and 50 µM).

| **Males** | | **TRABECULAR BONE** | | **CORTICAL BONE** | |
| --- | --- | --- | --- | --- | --- |
| ***Alcian blue Alizarin red staining*** | **Fgf20 KO** | 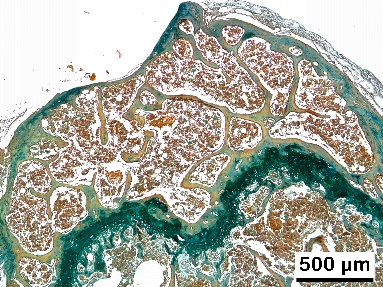 | 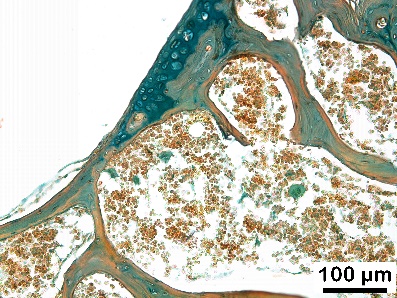 | 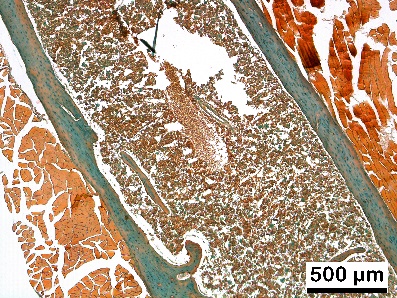 | 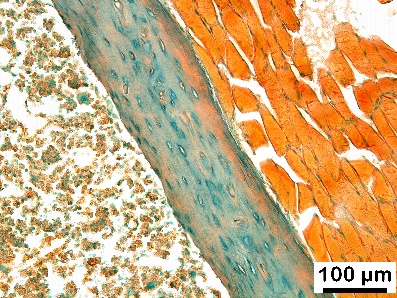 |
|  | **WT** | 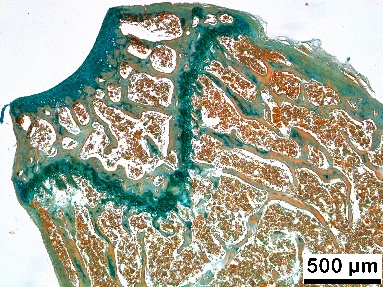 | 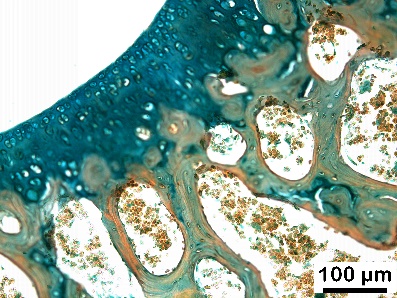 | 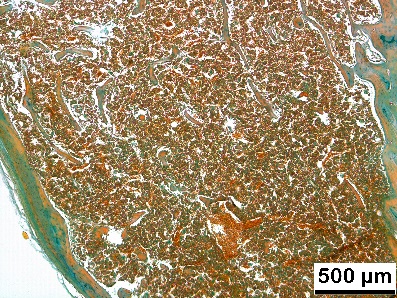 | 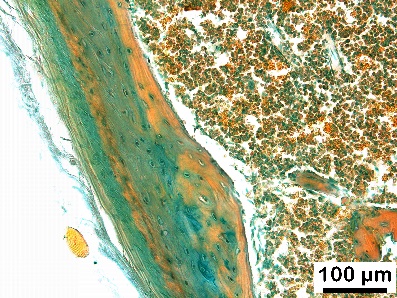 |
| ***Masson’s Trichrome staining*** | **Fgf20 KO** | 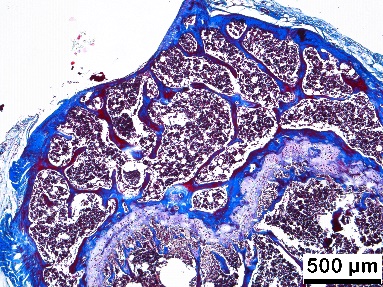 | 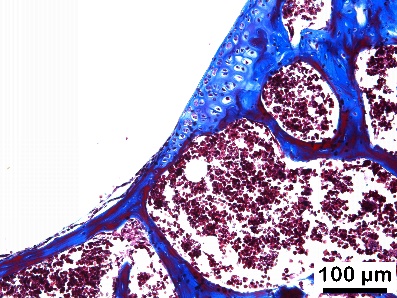 | 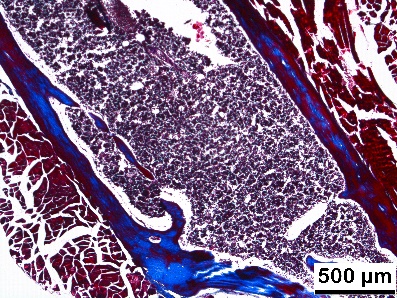 | 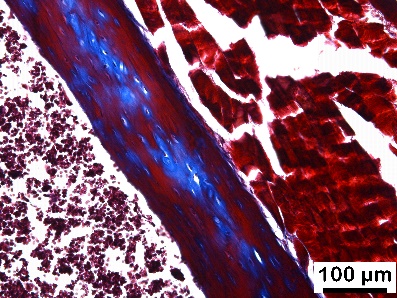 |
|  | **WT** | 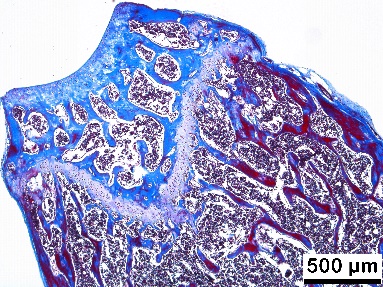 | 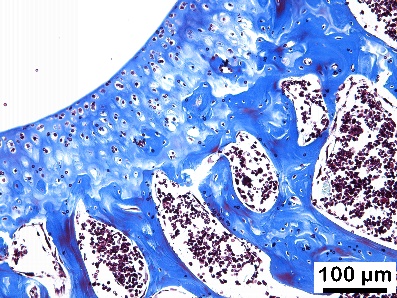 | 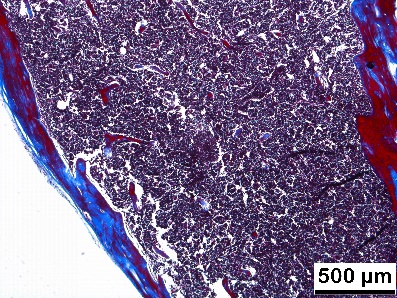 | 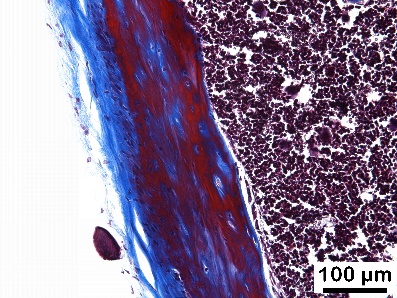 |
| ***TRAP staining*** | **Fgf20 KO** | 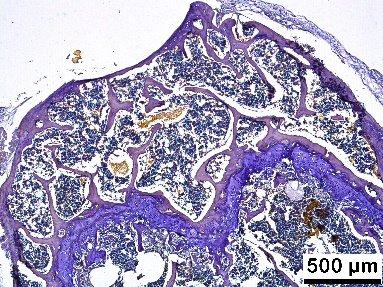 | 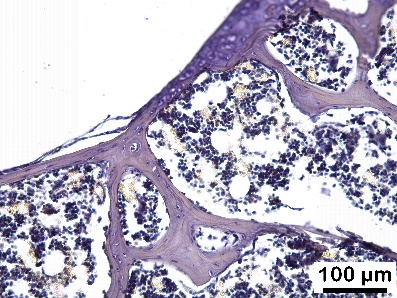 | 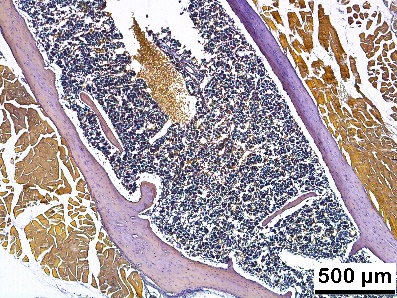 | 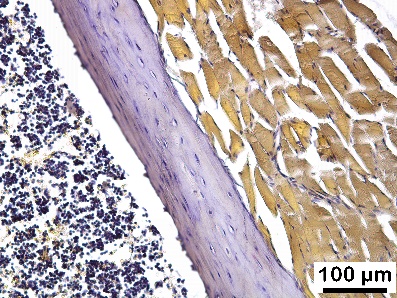 |
|  | **WT** | 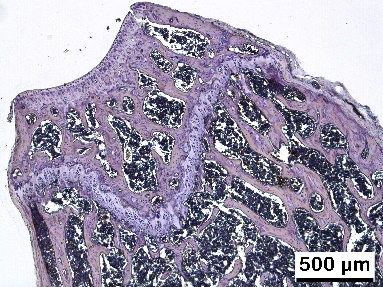 | 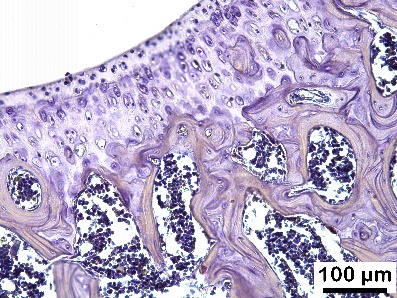 | 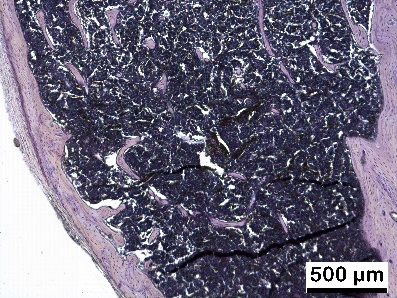 | 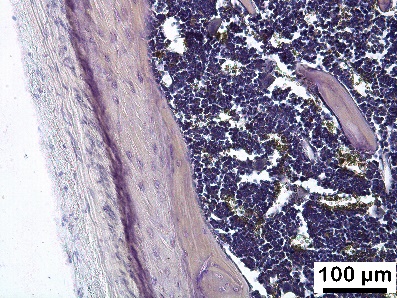 |

**Figure S2: Histological staining of male femur sections.** Alcian blue / Alizarin red, Masson’s Trichrome and TRAP staining were employed.

| **Females** | | **TRABECULAR BONE** | | **CORTICAL BONE** | |
| --- | --- | --- | --- | --- | --- |
| ***Alcian blue Alizarin red staining*** | **Fgf20 KO** | 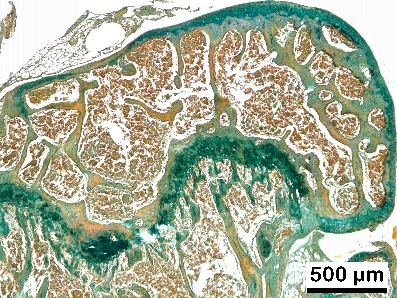 | 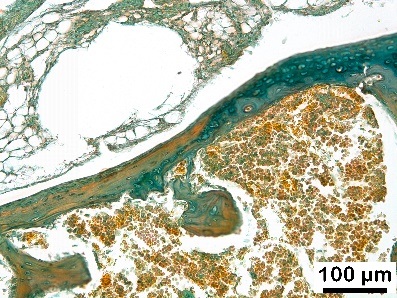 | 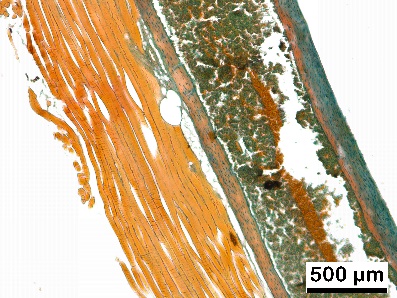 | 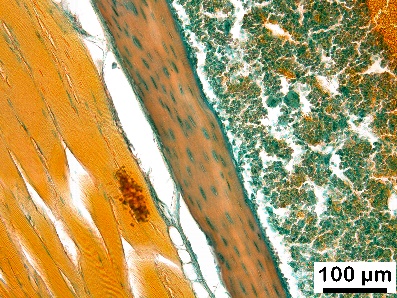 |
|  | **WT** | 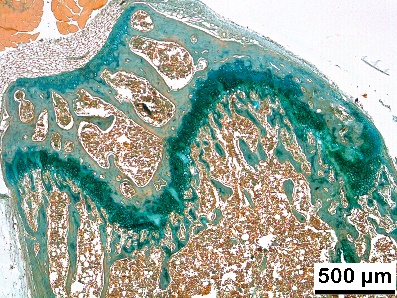 | 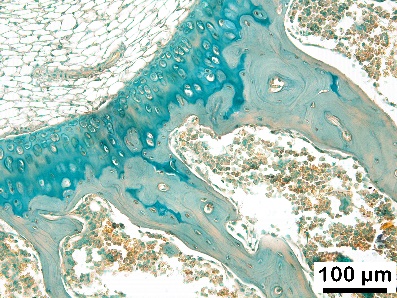 | 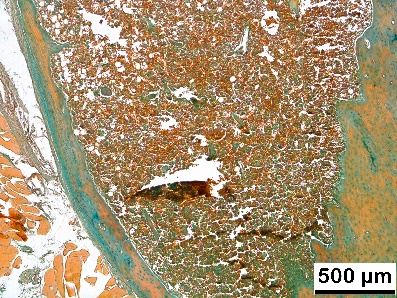 | 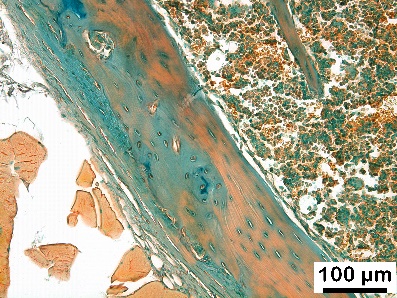 |
| ***Masson’s Trichrome staining*** | **Fgf20 KO** | 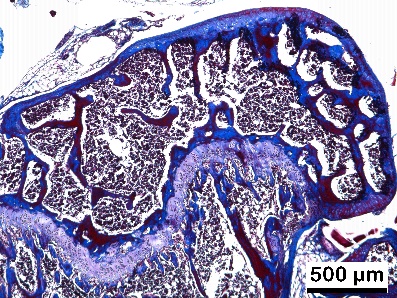 | 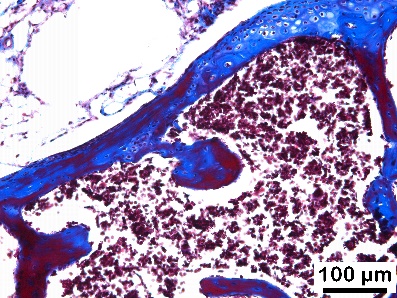 | 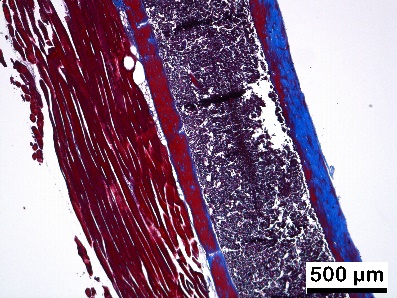 | 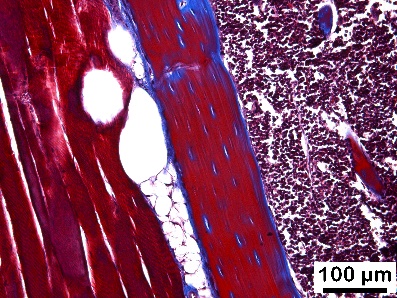 |
|  | **WT** | 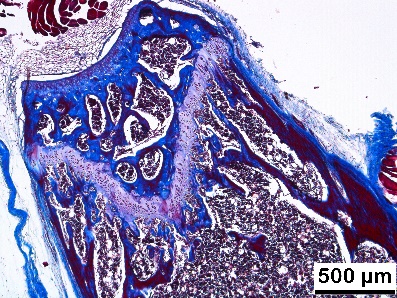 | 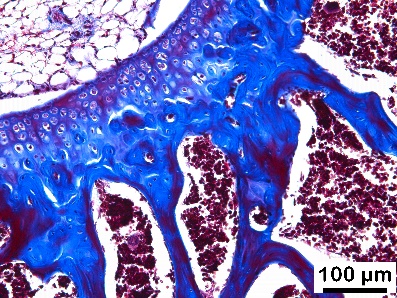 | 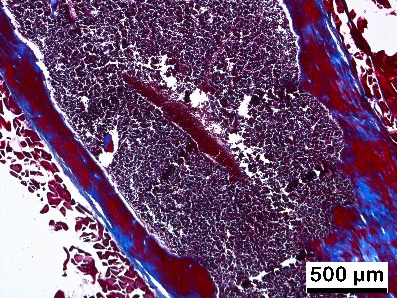 | 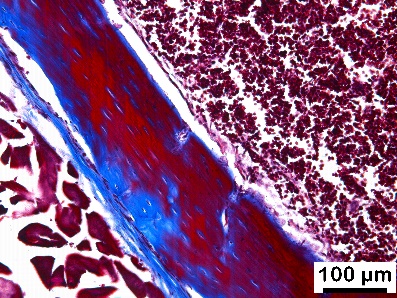 |
| ***TRAP staining*** | **Fgf20 KO** | 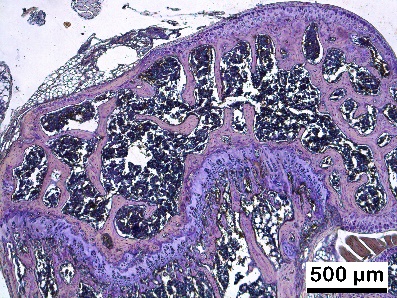 | 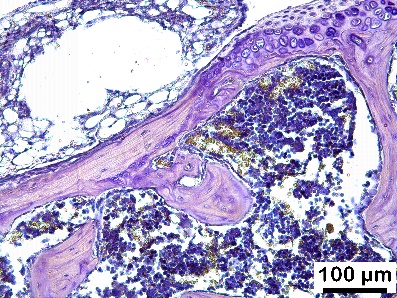 | 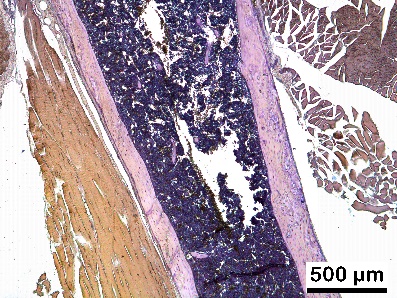 | 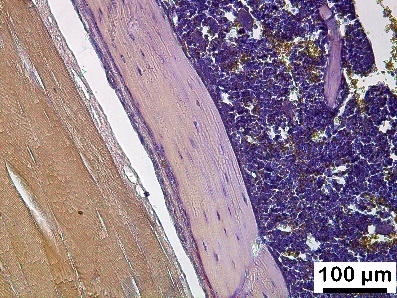 |
|  | **WT** | 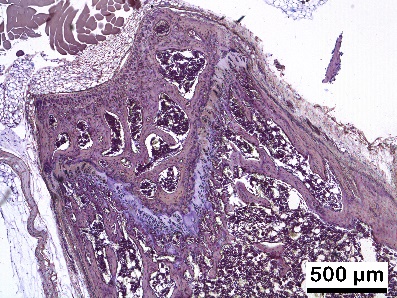 | 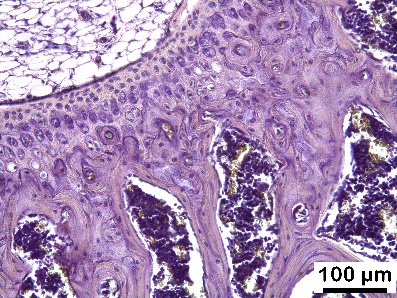 | 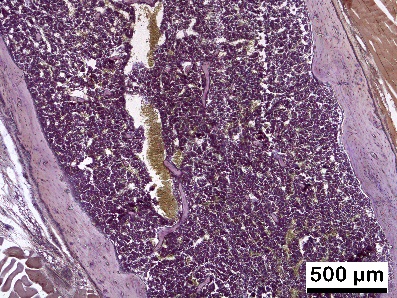 | 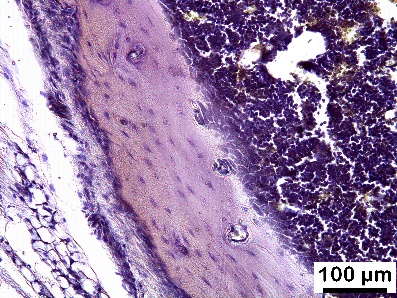 |

**Figure S3: Histological staining of female femur sections.** Alcian blue / Alizarin red, Masson’s Trichrome and TRAP staining were employed.
